# Generative emulation of weather forecast ensembles with diffusion models

**DOI:** 10.1126/sciadv.adk4489

**Published:** 2024-03-29

**Authors:** Lizao Li, Robert Carver, Ignacio Lopez-Gomez, Fei Sha, John Anderson

**Affiliations:** Google Research, Mountain View, CA, USA.

## Abstract

Uncertainty quantification is crucial to decision-making. A prominent example is probabilistic forecasting in numerical weather prediction. The dominant approach to representing uncertainty in weather forecasting is to generate an ensemble of forecasts by running physics-based simulations under different conditions, which is a computationally costly process. We propose to amortize the computational cost by emulating these forecasts with deep generative diffusion models learned from historical data. The learned models are highly scalable with respect to high-performance computing accelerators and can sample thousands of realistic weather forecasts at low cost. When designed to emulate operational ensemble forecasts, the generated ones are similar to physics-based ensembles in statistical properties and predictive skill. When designed to correct biases present in the operational forecasting system, the generated ensembles show improved probabilistic forecast metrics. They are more reliable and forecast probabilities of extreme weather events more accurately. While we focus on weather forecasting, this methodology may enable creating large climate projection ensembles for climate risk assessment.

## Teaser

Generative artificial intelligence technology accelerates ensemble weather forecasting at a fraction of the computational cost.

## INTRODUCTION

Weather is inherently uncertain, making the assessment of forecast uncertainty a vital component of operational weather forecasting (*1*, *2*). Given a numerical weather prediction (NWP) model, the standard way to quantify this uncertainty is to stochastically perturb the model’s initial conditions and its representation of small-scale physical processes to create an ensemble of weather trajectories (*3*). These trajectories are then regarded as Monte Carlo samples of the underlying probability distribution of weather states.

Given the computational cost of generating each ensemble member, weather forecasting centers can only afford to generate 10 to 50 members for each forecast cycle (*4*–*6*). This limitation is particularly problematic for users concerned with the likelihood of high-impact rare weather events, which typically require much larger ensembles to assess (*6*–*10*). For instance, one would need a 10,000-member calibrated ensemble to forecast the likelihood of events with 1% probability of occurrence with a relative error less than 10%. Large ensembles are even more necessary for forecasting compound extreme events (*11*). Extended-range and convective-scale forecasting, among other NWP applications, would also benefit from access to larger ensembles (*12*, *13*). Besides relying on increases in available computational power to generate larger ensembles in the future, it is imperative to explore more efficient approaches for generating ensemble forecasts.

In this context, recent advances in generative artificial intelligence (GAI) offer a potential path toward massive reductions in the cost of ensemble forecasting. GAI models extract statistical priors from datasets and enable conditional and unconditional sampling from the learned probability distributions. Through this mechanism, GAI techniques reduce the cost of ensemble forecast generation: Once learning is complete, the sampling process is far more computationally efficient than time-stepping a physics-based NWP model.

In this work, we propose a technique that is based on probabilistic diffusion models, which have recently revolutionized GAI use cases such as image and video generation (*14*–*16*). This technique is also gaining traction in weather and climate applications such as nowcasting (*17*, *18*) and upsampling (*19*, *20*). Our Scalable Ensemble Envelope Diffusion Sampler (SEEDS) can generate an arbitrarily large ensemble conditioned on as few as one or two forecasts from an operational NWP system. We compare the generated ensembles to ground-truth ensembles from the operational systems and to ECMWF Reanalysis v 5 (ERA5) (*21*). The generated ensembles not only yield weather-like forecasts but also match or exceed physics-based ensembles in skill metrics such as the rank histogram, the root mean squared error (RMSE), and the continuous ranked probability score (CRPS). In particular, the generated ensembles assign more accurate likelihoods to the tail of the forecast distribution, such as ±2σ and ± 3σ weather events. The computational cost of the model is negligible; it has a throughput of 256 ensemble members (at 2° resolution) per 3 min on Google Cloud TPUv3-32 instances and can easily scale to higher throughput by deploying more accelerators. We apply our methodology to uncertainty quantification in weather forecasting due to the wealth of data available and the ability to validate models on reanalysis. Nevertheless, the same approach could be used to augment climate projection ensembles.

Previous work leveraging artificial intelligence to augment and postprocess ensemble or deterministic forecasts has focused on improving the aggregate output statistics of the prediction system. Convolutional neural networks have been used to learn a global measure of forecast uncertainty given a single deterministic forecast, trained using as labels either the error of previous forecasts or the spread of an ensemble system (*22*). This approach has been generalized to predict the ensemble spread at each location of the input deterministic forecast, over both small regions using fully connected networks (*23*) or over the entire globe using conditional generative adversarial networks (*24*) based on the pix2pix architecture (*25*). Deep learning has also proved effective in calibrating limited-size ensembles. For instance, self-attentive transformers can be used to calibrate the ensemble spread (*26*). More related to our work, deep learning models have been successfully used to correct the probabilistic forecasts of ensemble prediction systems such that their final skill exceeds that of pure physics-based ensembles with at least double the number of members (*27*).

Our work differs from these previous studies in that our probabilistic generative model outputs high-dimensional weather-like samples from the target forecast distribution, akin to generative precipitation downscaling models (*28*). Thus, our approach offers added value beyond improved estimates of the ensemble mean and spread: The drawn samples can be used to characterize spatial patterns associated with weather extremes (*29*) or as input to targeted weather applications that depend on variable and spatial correlations (*7*).

## RESULTS

Using the SEEDS methodology, we have developed two generative models. The generative ensemble emulation (SEEDS-GEE) model learns to emulate the distribution of the U.S. operational ensemble NWP system, the Global Ensemble Forecast System (GEFS) version 12 (*5*). The generative postprocessing (SEEDS-GPP) model learns to emulate a blended distribution that combines the GEFS ensemble with historical data from the ERA5 reanalysis of the European Centre for Medium-Range Weather Forecasts (ECMWF), aiming to correct underlying biases in the operational GEFS system (i.e., postprocessing). The justification for blending distributions is explained in the Supplementary Materials.

SEEDS-GEE is trained using 20 years of GEFS 5-member retrospective forecasts (*30*), and SEEDS-GPP additionally learns from ECMWF’s ERA5 10-member reanalysis ensemble over the same period (*21*). Once learned, both models take as inputs a few randomly selected member forecasts from the operational GEFS ensemble, which has 31 members. We refer to the selected members as the seeding forecasts. These seeds provide the physical basis used by the generative models to conditionally sample additional plausible weather states. Both SEEDS-GEE and SEEDS-GPP can be used to generate ensembles with a substantially larger number of forecasts than operational physics-based systems, easily reaching hundreds to tens of thousands of members.

**Fig. 1.**
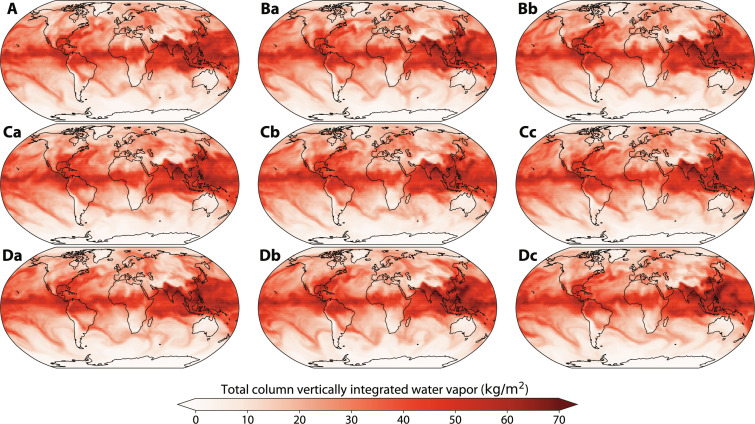
Global chart of total column vertically integrated water vapor on 14 July 2022 at 0:00 UTC. (**A**) The ERA5 reanalysis. (**Ba** and **Bb**) Two members from the 7-day GEFS-Full forecasts used as seeds. (**Ca** to **Cc**) Three samples from SEEDS-GEE. (**Da** to **Dc**) Three nonseeding members from the 7-day GEFS-Full forecasts.

Figure 1 compares samples from the GEFS operational system, the ERA5 reanalysis, and the generative emulator SEEDS-GEE. We also assess the quality of the generated ensembles in terms of multiple important characteristics of useful ensemble prediction systems. First, we analyze whether the forecasts in the generative ensembles display spatial coherence, multivariate correlation structures, and wave number spectra consistent with actual weather states. Second, we compare the pointwise predictive skill of the generative ensembles and the full operational physics-based GEFS ensemble, measured against the ERA5 high-resolution (HRES) deterministic reanalysis (*21*).

We report results on a subset of field variables: the mean sea level pressure, the temperature at 2 m, and the zonal wind speed at a pressure level of 850 hPa. Results for all modeled fields, listed in Table 1, are presented in section S3.2. We use GEFS-Full to refer to the full 31-member GEFS ensemble and GEFS-2 to an ensemble made of two randomly selected seeding forecasts. Unless noted, our generated ensembles have 512 members.

**Table 1.** List of atmospheric state variables that are modeled.

Quantity	Processed units
Mean sea level pressure	Pa
Temperature at 2 m	K
Eastward wind speed at 850 hPa	m/s
Northward wind speed at 850 hPa	m/s
Geopotential at 500 hPa	m^2^/s^2^
Temperature at 850 hPa	K
Total column water vapor	kg/m^2^
Specific humidity at 500 hPa	kg/kg

### Generated weather forecasts are plausible weather maps

Ensemble forecasting systems are most useful when individual weather forecasts resemble real weather maps (*31*). This is because for many applications, such as ship routing, energy forecasting, or compound extreme event forecasting, capturing cross-field and spatial correlations is fundamental (*7*, *29*, *32*).

**Fig. 2.**
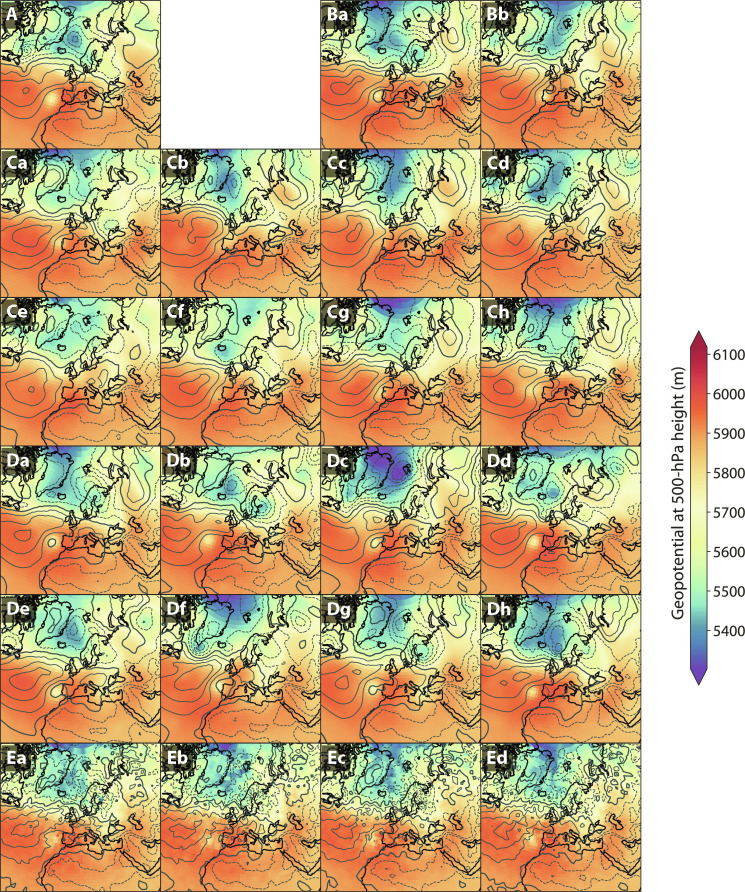
Stamp maps over Europe on 14 July 2022 at 0:00 UTC. The contours are for the mean sea level pressure (dashed lines mark isobars below 1010 hPa), while the heatmap depicts the geopotential height at the 500-hPa pressure level. (**A**) The ERA5 reanalysis. (**Ba** and **Bb**) Two members from the 7-day GEFS-Full forecasts used as seeds. (**Ca** to **Ch**) Eight samples drawn from SEEDS-GEE. (**Da** to **Dh**) Eight nonseeding members from the 7-day GEFS-Full forecasts. (**Ea** to **Ed**) Four samples from a pointwise Gaussian model parameterized by the mean and variance of the entire GEFS-Full ensemble.

To investigate this aspect of weather forecasts, we compare the covariance structure of the generated samples to those from the ERA5 reanalysis and GEFS through a stamp map over Europe for a date during the 2022 European heatwave in Fig. 2 (*33*). The global atmospheric context of a few of these samples is shown in Fig. 1 for reference. We also present in Fig. 2 weather samples obtained from a Gaussian model that predicts the univariate mean and SD of each atmospheric field at each location, such as the data-driven model proposed in (*24*). This Gaussian model is meant to characterize the output of pointwise postprocessing (*22*–*24*), which ignores correlations and treats each grid point as an independent random variable.

SEEDS-GEE captures well both the spatial covariance and the correlation between mid-tropospheric geopotential and mean sea level pressure because it directly models the joint distribution of the atmospheric state. The generative samples display a geopotential trough west of Portugal with spatial structure similar to that found in samples from GEFS-Full or the reanalysis. They also depict realistic correlations between geopotential and sea level pressure anomalies. Although the Gaussian model predicts the marginal univariate distributions adequately, it fails to capture cross-field or spatial correlations. This hinders the assessment of the effects that these anomalies may have on hot air intrusions from North Africa, which can exacerbate heatwaves over Europe (*34*).

**Fig. 3.**
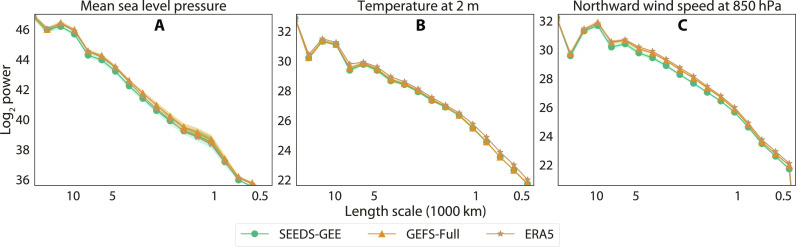
The energy spectra of several global atmospheric variables for the month of January of 2022. For ensemble forecasts GEFS-Full and SEEDS-GEE, the line shows the mean of the spectra of the members and the shading shows the upper and lower extremes of the spectra among all the members for forecasts with a 7-day lead time. GEFS-Full has 31 members, and SEEDS-GEE has 512 samples generated conditioned on 2 of the 31 members. The panels are for different variables. (**A**) Mean sea level pressure. (**B**) Temperature at 2 m. (**C**) Northward wind speed at 850 hPa.

Figure 3 contrasts the energy spectra of SEEDS-GEE forecasts with that of ERA5 and GEFS-Full (see section S3.1.7 for more details). The large overlap between samples from both forecast systems and the reanalysis demonstrates that the two ensembles have similar energies at all length scales. Small systematic differences can be observed in some variables such as the meridional wind in the low troposphere, but for most variables, the differences between SEEDS-GEE and GEFS-Full are similar to the differences between the operational system and the ERA5 reanalysis.

**Fig. 4.**
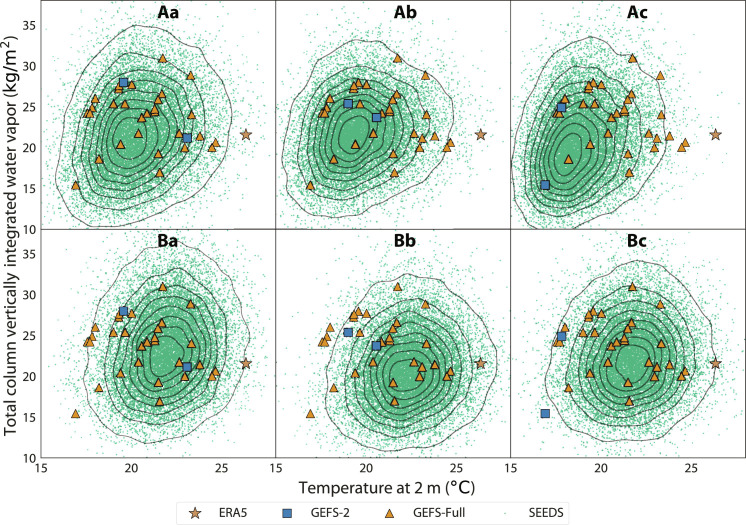
Generated ensembles provide better statistical coverage of the 14 July 2022 European extreme heat event. Each plot shows the values of two variables over a grid point near Lisbon, Portugal from 16,384 samples generated by our models conditioned on two seeds taken from the 7-day GEFS-Full ensemble forecasts. The valid forecast time is 1:00 local time. The solid contour levels correspond to iso-proportions of the kernel density of SEEDS, with the outer most one encircling 95% of the mass and 11.875% between each level. In particular, (**Aa**) to (**Ac**) show SEEDS-GEE samples conditioned on three different sets of random seeds, while (**Ba**) to (**Bc**) show SEEDS-GPP in the same way.

In addition to examining the coherence of regional structures and the global spectra of the generative samples, we also examine the multivariate correlation structure of generative samples locally. Figure 4 depicts the joint distributions of temperature at 2 m and total column water vapor at the grid point covering Lisbon during the extreme heat event on 14 July 2022 at 1:00 local time. We used the 7-day forecasts issued on 7 July 2022. For each plot, we generate 16,384-member ensembles. The observed weather event from ERA5 is denoted by the star. The operational ensemble is also shown, with squares denoting the forecasts used to seed the generated ensembles and triangles denoting the rest of the GEFS ensemble members.

According to the GEFS-Full ensemble, the observed event was so unlikely 7 days prior that none of its members attained near-surface temperatures as warm as those observed. The event probability computed from the GEFS-Full Gaussian kernel density estimate is lower than 1%, which means that ensembles with less than 100 members are unlikely to contain forecasts as extreme as this event. In contrast, the generated ensembles are able to extrapolate from the two seeding forecasts, providing an envelope of possible weather states with much better statistical coverage of the event. This allows both quantifying the probability of the event taking place and sampling weather regimes under which it would occur. Specifically, our highly scalable generative approach enables the creation of very large ensembles that can characterize very rare events by providing samples of weather states exceeding a given threshold for any user-defined diagnostic.

Moreover, we observe that the distributions of the generated ensembles do not depend critically on the (positioning of the) seeding forecasts. This suggests that the generative approach is plausibly learning the intrinsic dynamical structure, i.e., the attractor of the atmosphere, to expand the envelopes of the phase of the dynamical systems to include extreme events that deviate strongly from the seeds. For instance, it is possible that other variables might be able to provide valuable cues to capture the distributions of the plotted two variables.

### Forecast reliability and predictive skill

An important characteristic of ensemble forecast systems is their ability to adequately capture the full distribution of plausible weather states. This characteristic is known as forecast calibration or reliability (*35*). Forecast reliability can be characterized for a given lead time in terms of the rank histogram (*36*, *37*). Deviations from flatness of this histogram indicate systematic incompatibility between the ensemble forecast distribution and the observations. The bulk deviation from flatness can be used as an unreliability metric δ (*38*), defined in section S3.1, such that higher values of δ indicate a lower reliability of the forecasts.

**Fig. 5.**
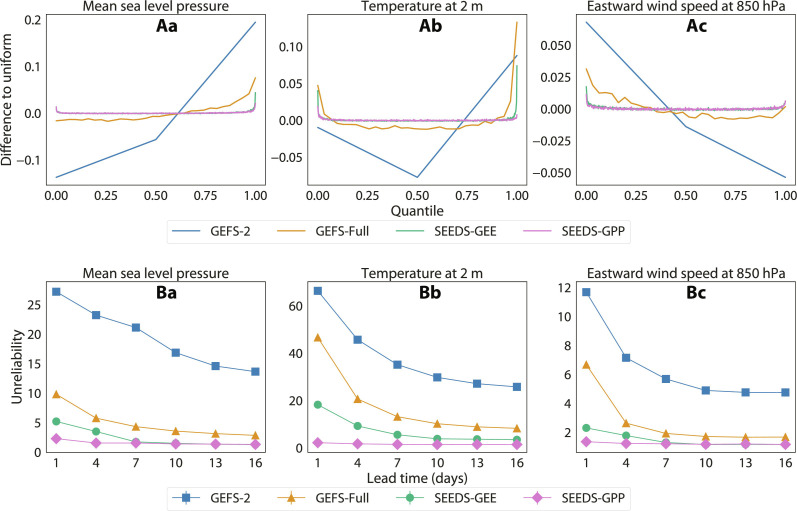
Rank histograms and unreliability indices of the generated samples. (**A**) Rank histograms from 7-day forecasts for the grid points in the region bounded by parallels 34 N and 42 N and meridians 124 W and 114 W for the year 2022. This region roughly encompasses California and Nevada, USA. To compare the histograms of ensembles of different size, the *x* axis is normalized to quantiles instead of ranks, and the *y* axis shows the difference to the uniform distribution. A perfectly calibrated ensemble forecast should have a flat line at 0. (**B**) The unreliability index δ (*37*) for forecast ensembles at different lead times computed from global data. In both cases, ERA5 for the entire 2022 is the ground truth, and both SEEDS-GEE and SEEDS-GPP have 512 samples generated from two random seeds GEFS-2 from the GEFS-Full. The columns are for (**a**) mean sea level pressure, (**b**) 2-m temperature, and (**c**) eastward wind speed at 850 hPa.

Rank histograms for 7-day forecasts from GEFS-Full, GEFS-2, SEEDS-GEE, and SEEDS-GPP over California and Nevada are shown in the top row of Fig. 5. The GEFS ensembles display systematic negative biases in mean sea level pressure and near-surface temperature over the region, a positive bias in zonal wind speed at 850 hPa, and an underestimation of near-surface temperature uncertainty. Our model ensembles have better resolution at estimating forecast probabilities due to the larger number of members that can be effortlessly generated. This results in better reliability than GEFS-Full. In addition, SEEDS-GPP substantially reduces the ensemble underdispersion for near-surface temperature forecasts and reduces the biases in the other two variables, validating SEEDS-GPP as a useful debiasing methodology. Reliability diagrams for all models and different percentiles are included in fig. S9.

The improved reliability of the generated ensembles is consistent on a global scale and for all lead times, as shown by the unreliability metric δ in the bottom row of Fig. 5. SEEDS-GPP attains the highest reliability of all ensembles, most noticeably in the first forecast week. Values of δ for different generated ensemble sizes are included in fig. S10.

The predictive skill of the generated ensembles is measured in terms of the RMSE and the anomaly correlation coefficient (ACC) of the ensemble mean, as well as the CRPS, treating the ERA5 HRES reanalysis as the reference ground truth. These metrics are computed and averaged over the grid points every forecast day in the test set and then aggregate over the test days. Section S3.1 details how these metrics are defined.

**Fig. 6.**
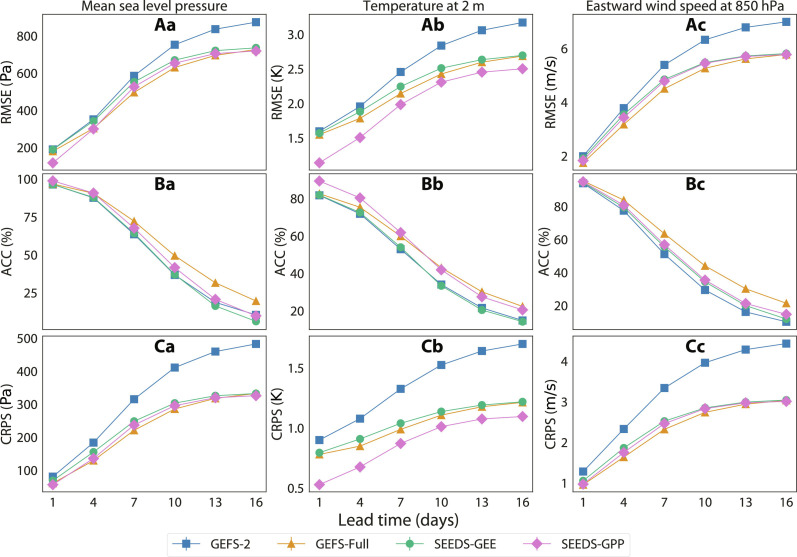
Point-wise skill of the generative ensembles. Global data from ERA5 for the entire 2022 are the ground truth. Both SEEDS-GEE and SEEDS-GPP have 512 samples generated from two random seeds GEFS-2 from the GEFS-Full ensemble. The three rows correspond to the metrics (**A**) RMSE of the ensemble mean, (**B**) ACC of the ensemble mean, and (**C**) CRPS of the ensemble. The columns are for variables (**a**) mean sea level pressure, (**b**) 2-m temperature, and (**c**) eastward wind speed at 850 hPa.

Figure 6 reports these metrics for three atmospheric fields: the mean sea level pressure, the temperature 2 m above the ground, and the eastward wind speed at 850 hPa. Both SEEDS-GEE and SEEDS-GPP perform substantially better than the seeding GEFS-2 ensemble across all metrics. The emulator SEEDS-GEE shows similar but slightly lower skill than GEFS-Full across all metrics and variables. Our SEEDS-GPP is noticeably better than the physics-based GEFS-Full at predicting near-surface temperature, roughly matching its skill for the other two fields. Intuitively, the potential benefits of statistical blending with a corrective data source are determined by the variable-dependent biases of the emulated forecast model. In this case, the GEFS model is known to have a cold bias near the surface (*5*).

**Fig. 7.**
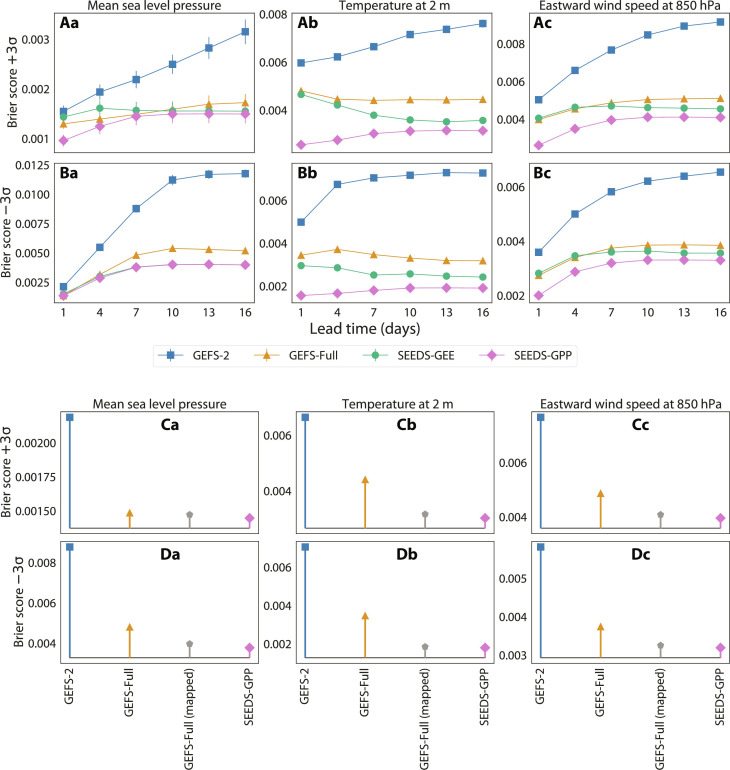
Binary classification skill on extreme events. Global data from ERA5 for the entire 2022 are the ground truth. Both SEEDS-GEE and SEEDS-GPP have 512 samples generated from two random seeds GEFS-2 from the GEFS-Full ensemble. The top two rows show the Brier score at different lead times for (**A**) events +3σ and (**B**) −3σ from climatology, while (**C** and **D**) the bottom two rows compare SEEDS-GPP to statistical postprocessing of the GEFS-Full ensemble through quantile mapping, for 7-day lead time forecasts. Lower Brier scores are better. The columns are for (**a**) mean sea level pressure, (**b**) 2-m temperature, and (**c**) eastward wind speed at 850 hPa.

A particularly challenging but important task of ensemble forecasts is being able to forecast extreme events and assign meaningful likelihoods to them (*7*). Figure 7 compares the skill of the same four ensembles in predicting events deviating at least ±3σ from the mean climatology. We measure binary classification skill by computing the Brier score of occurrences using ERA5 HRES as the binary reference and assigning a probability of occurrence to the ensemble forecasts equal to the fraction of occurrences within the ensemble.

We observe that SEEDS-GEE has slightly higher skill than the full ensemble GEFS-Full and far exceeds the skill of the seeding forecast ensemble GEFS-2, particularly at long lead times. In the forecast of temperature at 2 m and zonal wind speed, SEEDS-GPP performs noticeably better than the other ensembles. For other variables, despite the less apparent advantage, SEEDS-GPP remains the best extreme forecast system for most lead times. This highlights the relevance of our generative approach for forecasting tasks focused on extremes.

The bottom rows of Fig. 7 further compare SEEDS-GPP to quantile mapping, a statistical postprocessing method described in section S3.4. SEEDS-GPP still matches or outperforms the postprocessed GEFS-Full ensemble in the prediction of extreme events, although the generative approach only requires two physics-based forecasts. Comparisons for additional metrics are included in fig. S15. We note that statistical postprocessing, as opposed to SEEDS-GPP, is an application-specific downstream task that could also be applied to the emulator ensembles.

### Hallucination or in-filling?

One frequently cited issue of generative AI technology is its tendency to “hallucinate information.” We conclude this section by exploring the nature of the distribution information that the generative ensembles are able to represent, beyond what is present in the two seeding forecasts from the GEFS full ensemble. As shown previously, the generated ensembles outperform the seeding forecast ensembles in all metrics and often match or improve over the full physics-based ensembles.

**Fig. 8.**
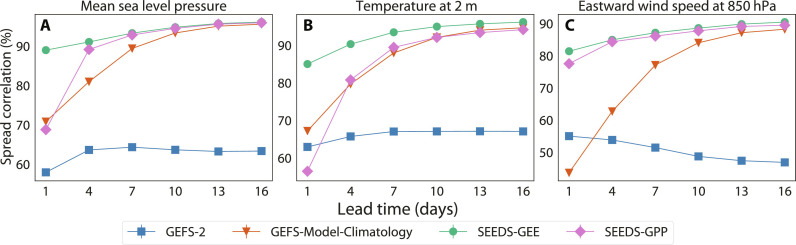
Comparison of the ensemble spread correlation between the generated ensembles and physical forecast ensembles. Global data from the GEFS-Full ensemble for the entire 2022 are the ground truth to correlate against. Both SEEDS-GEE and SEEDS-GPP have 512 samples generated from two random seeds GEFS-2. The GEFS-Model-Climatology ensemble spread is computed per lead time and day of year from the GEFS reforecast dataset. Details can be found in section S3.1.6. The three panels are for (**A**) mean sea level pressure, (**B**) 2-m temperature, and (**C**) eastward wind speed at 850-hPa pressure level.

Figure 8 measures the correlation of the generative ensembles (SEEDS-GEE and SEEDS-GPP), the seeding ensemble GEFS-2, and the GEFS model climatology, with respect to the GEFS-Full ensemble forecasts. While comparing full joint distributions remains infeasible, we compute how well the spread, which is the pointwise ensemble SD, of each ensemble forecast correlates with that of the full physics-based ensemble GEFS-Full. The plots show that at long-lead times (≥10 days), all ensembles but GEFS-2 converge to high correlations (≥90%) with GEFS-Full. This is also true for the model climatology. However, in the medium range (more than 4 days but less than 10 days ahead), the generative ensembles display a higher correlation with the GEFS-Full than both the model climatology and GEFS-2. This suggests that the generative models are indeed able to generate information about forecast uncertainty beyond the two seeding forecasts.

In addition, the fact that generative ensembles can capture a higher correlation with GEFS-Full than the model climatology in the short and medium ranges shows that the diffusion models are learning to emulate dynamically relevant features beyond model biases; they have resolution beyond climatology. Thus, we put forward a reasonable hypothesis that the generated ensembles in-fill probability density gaps in the small seeding ensembles. They also extend the (tails of the) envelopes of the full ensembles such that rare events are well represented in the envelopes.

This analysis and the results shown in Figs. 2 and 3 show that the generated samples correlate with trajectories of the emulated model and display a similar spectra, spatial coherence, and variable correlations. Nevertheless, the occurrence of hallucinations in individual samples cannot be ruled out by these diagnostics. Frameworks are rapidly being developed that will hopefully enable more detailed assessments of their physical consistency in the near future (*39*, *40*).

## DISCUSSION

The SEEDS proposed in this work leverages the power of GAI to produce ensemble forecasts comparable to those from the operational GEFS system at accelerated pace—the results reported in this paper need only 2 seeding forecasts from the operational system, which generates 31 forecasts in its current version (*5*). This leads to a hybrid forecasting system where a few weather trajectories computed with a physics-based model are used to seed a diffusion model that can generate additional forecasts much more efficiently. This methodology provides an alternative to the current operational weather forecasting paradigm, where the computational resources saved by the statistical emulator could be allocated to increasing the resolution of the physics-based model (*41*) or issuing forecasts more frequently (*42*).

SEEDS is trained on historical retrospective forecasts (i.e., reforecasts) issued with the operational physics-based model, which are already required for postprocessing in the current paradigm (*43*). Our framework is also flexible enough to enable direct generation of debiased ensembles when the generative postprocessing task is considered during training; the only additional requirement is access to historical reanalysis for the reforecast period.

For future work, we will conduct case studies of high-impact weather events to further evaluate SEEDS’s performance and consider specific ensemble forecast applications such as tropical and extratropical cyclone tracking (*8*, *44*). We will also explore more deeply the statistical modeling mechanisms that these models use to extract information from weather data and in-fill the ensemble forecast distribution. It is our belief that our application of generative AI to weather forecast emulation represents just one way of many that will accelerate progress in operational NWP in coming years. In addition, we hope the established utility of generative AI technology for weather forecast emulation and postprocessing will spur its application in research areas such as climate risk assessment, where generating a large number of ensembles of climate projections is crucial to accurately quantifying the uncertainty about future climate (*10*, *45*).

## MATERIALS AND METHODS

We start by framing the learning tasks. We then outline the data and neural network learning algorithm we use. Details, including background, data processing and preparation, and learning architectures and procedures, are presented in sections S1 and S2.

### Setup

To address the computational challenge of generating large weather forecast ensembles, we consider two learning tasks: generative ensemble emulation and generative postprocessing. In both tasks, we are given as inputs a few examples sampled from a probability distribution *p*(***v***), where ***v*** stands for the atmospheric state variables. In our case, these examples represent physics-based weather forecasts. We seek to generate additional samples that either approximate the same distribution or a related desired distribution. The central theme of statistical modeling for both tasks is to construct a computationally fast and scalable sampler for the target distributions.

Generative ensemble emulation leverages *K* input samples to conditionally generate *N > K* samples such that they approximate the original distribution *p*(***v***) from which the input samples are drawn. Its main purpose is to augment the ensemble size inexpensively without the need to compute and issue more than *K* physics-based forecasts.

In generative postprocessing, the sampler generates *N > K* samples such that they approximate a mixture distribution where *p*(***v***) is just one of the components. We consider the case where the target distribution is α *p*(***v***) *+* (*1* − α) *p*′(***v***), with 0 ≤ α < 1 being the mixture weight and *p*′(***v***) a different distribution. The generative postprocessing task aims not only to augment the ensemble size but also to bias the new samples toward *p*′(***v***), which we take to be a distribution that more closely resembles actual weather. The underlying goal is to generate ensembles that are less biased than those provided by the physics-based model, while still quantifying the forecast uncertainty captured by *p*(***v***). We emphasize that while this task has the flavor and also achieves the effect of debiasing to some degree, we focus on generating samples instead of minimizing the difference between their mean and a given reanalysis or observations. In both the emulation and postprocessing tasks, the smaller the value of *K* is, the greater the computational savings.

**Fig. 9.**
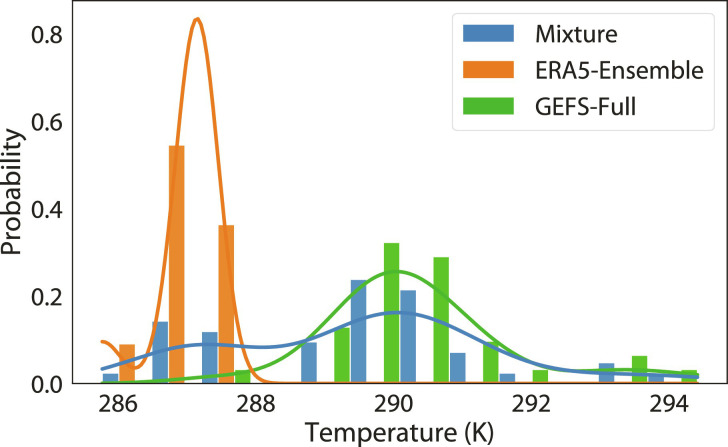
Illustration of the target distributions of generative ensemble emulation and postprocessing. The plot shows empirical distributions of the surface temperature near Mountain View, CA on 4 July 2021 in the GEFS and ERA5 ensembles, with the bars for the histograms with 12 shared bins and the curves for the corresponding Gaussian kernel density estimators.

Figure 9 illustrates the concepts behind these two tasks. There, *p*(***v***) is the distribution of the surface temperature near Mountain View, CA on 4 July 2021 as predicted by the GEFS 13-day forecast ensemble (*5*), and *p*′(***v***) is the corresponding ERA5 reanalysis ensemble (*21*). While the GEFS ensemble has 31 members, our goal is to use *K* ≪ 31 GEFS ensemble members to steer our samplers to generate additional forecast members that are consistent with either GEFS’s statistics or the mixture distribution’s statistics. Inspired by terminology from natural language understanding and computer vision, we refer to those *K* input examples from *p*(***v***) as “seeds.” The desirability to have a small *K* is in spirit similar to few-shot learning setups in those works.

We stress that the primary goal of both tasks is to improve the computational efficiency of ensemble weather forecasting, not to replace physics-based models. The generated samples should be not only consistent with the underlying distribution of atmospheric states (each sample is “weather-like”) but also validated by standard forecast verification metrics. In this work, we examine the generated ensembles by comparing them to other physics-based ensembles using the rank histogram, the ACC, RMSE, CRPS, and rare event classification metrics, as defined in section S3.

### Data for learning and evaluation

We target the GEFS (version 12) ensemble forecasting system for the generative ensemble emulation task (*5*). We use 20 years of GEFS five-member reforecasts (*30*), denoted hereafter as GEFS-RF5, to learn *p*(***v***). We favor the use of reforecasts over past operational forecasts because they provide data from GEFS version 12 spanning a longer time range and sampling more weather regimes (*30*, *46*); operational forecasts from this version of the model only started in 2020 (*5*). Generative postprocessing attempts to remove systematic biases of the original forecasting system from the learned emulator. To this end, we take the ERA5 10-member reanalysis ensemble (*21*), denoted as ERA5-10, to represent *p*′(***v***) in the target mixture distribution. We also use the ERA5 HRES as a proxy for real historical observations when evaluating the skill of our generated ensemble predictions.

All data are derived from publicly available sources. In particular, we use GEFS-RF5 (*30*) and ERA5-10 (*21*) from 1 January 2000 to 31 December 2019 for training. GEFS-Full (*5*) and ERA5-HRES (*2*) from 23 September 2020 to 31 December 2021 are used for validation, while the entire year 2022 data from these two sources are reserved for evaluation. Table 1 lists the atmospheric state variables that are considered by our models. We model continuous, meteorologically relevant variables that exhibit approximately normal climatologies. In particular, we focus on variables characterizing conditions at the surface and the entire vertical column, just above the atmospheric boundary layer where daily fluctuations are smaller and in the mid-troposphere as representative of weather systems. We omit precipitation because the dataset we use for validation (ERA5) is known to have important biases in this field (*47*). All variables are extracted and spatially regridded to the same cubed sphere mesh with a size of 6 × 48 × 48 (about 2° resolution) using inverse distance weighting with four neighbors (*48*). We only retain the 00-hour UTC time snapshots of the fields in Table 1 for each day.

The climatology is computed from the ERA5 HRES dataset, using the reference period 1990–2020. The daily climatological mean and SD are obtained by smoothing these two timeseries with a 15-day centered window over the year with periodic boundary conditions. The mean and SD for 29th of February is the average of those for 28th February and 1st of March.

Our models take as inputs and produce as outputs the standardized climatological anomalies of variables in Table 1, defined as the standardized anomalies using the aforementioned climatological mean and SD for the day of year and location, which facilitates learning (*49*–*51*). In particular, the inputs to the model are *K* forecast anomalies and the climatological mean, which are used as conditional information for the diffusion model (see details in section S2.3). The outputs are converted back to raw values for evaluation.

For each unique pair of forecast lead time and number of seeds *K*, we train a diffusion model for the generative ensemble emulation task. For each unique triplet of lead time, *K*, and mixture weight α, we train a model for the generative postprocessing task. We provide results for lead times of 1, 4, 7, 10, 13, and 16 days, *K* = 2 seeds, and generated ensembles with *N* = 512 members. For the postprocessing task, we consider the mixing ratio α = 0.5. The sensitivity to *K*, *N*, and α is explored in section S3.3.

We evaluate our models against the operational GEFS 31-member ensemble (GEFS-Full) and the ERA5 HRES reanalysis. Note that we can do so because GEFS-Full and GEFS-RF5 are considered to have similar distributions—the reforecasts are reruns of the operational GEFS model using historical initial conditions (*30*). We use the 20 years from 2000 to 2019 for training, year 2020 and 2021 for validation, and year 2022 for evaluation. In particular, to accommodate the longest lead time of 16 days, we evaluate using the forecasts initialized from 1 January 2022 to 15 December 2022 (349 days in total) and the ERA5 HRES data aligned with the corresponding days.

### Learning method and architecture

The use of probabilistic diffusion models to parameterize the target distributions, conditioned on a few seeds, is at the core of our statistical modeling algorithm for both tasks. Probabilistic diffusion models are generative models of data. The generative process follows a Markov chain. It starts with a random draw from an initial noise distribution—often an isotropic multivariate Gaussian. Then, it iteratively transforms and denoises the sample until it resembles a random draw from the data distribution (*52*). The iteration steps advance the diffusion time, which is independent from the real-world time. The denoising operation relies on the instantiation of a diffusion time–dependent score function, which is the Jacobian of the log likelihood of the data at a given diffusion time (*53*). Score functions often take the form of deep learning architectures whose parameters are learned from training data.

Typically, the score is a function of the noisy sample and the diffusion time. In this case, the resulting data distribution is a model of the unconditional distribution of the training data. When additional inputs are passed to the score function, such as *K* seeding forecasts in our setting, the sampler constructs the distribution conditioned on these inputs.

In this work, our choice of the score function is inspired by the Vision Transformer (ViT), which has been successfully applied to a range of computer vision tasks (*54*). It is intuitive to view atmospheric data as a temporal sequence of snapshots, which are in turn viewed as “images.” Each snapshot is formed by “pixels” covering the globe with “color” channels. In this case, the channels correspond to the collection of atmospheric variables at different vertical levels. These can easily exceed in number the very few color channels of a typical image, e.g., three in the case of an red-green-blue image. Because of this, we use a variant of ViT via axial attention (*55*), so that the model remains moderate in size and can be trained efficiently.

Irrespective of the lead times and the number of seeds, all the models share the same architecture and have about 114 million trainable parameters. They are trained with a batch size of 128 for 200,000 steps. The training of each model takes slightly less than 18 hours on a 2 × 2 × 4 TPUv4 cluster. Inference (namely, ensemble generation) runs at batch size 512 on a 4 × 8 TPUv3 cluster at less than 3 min per batch. It is thus very efficient and easily scalable to generate thousands of members.

## Supplementary Materials

### This PDF file includes:

Supplementary Text Figs S1 to S15 References
