# Supplementary material for: Generative emulation of weather forecast ensembles with diffusion models
Source: Sci Adv. 2024 Mar 29;10(13):eadk4489. doi: 10.1126/sciadv.adk4489 (PMC10980268; doi:10.1126/sciadv.adk4489)
Supplement: Supplementary file 1 — Supplementary Text Figs S1 to S15 References [file sciadv.adk4489_sm.pdf]

Science Advances
AAAS

Supplementary Materials for

Generative emulation of weather forecast ensembles with diffusion models

Lizao Li *et al.*

Corresponding author: Fei Sha, fsha@google.com

Sci. Adv. **10**, eadk4489 (2024)
DOI: 10.1126/sciadv.adk4489

This PDF file includes:

Supplementary Text
Figs S1 to S15
References

Supplementary Text

S1 Probabilistic Diffusion Models

We give a brief introduction to probabilistic diffusion models. Diffusion models are powerful methods for learning distributions from data. They have recently become one of the most popular approaches for image generation and video synthesis [15]. For a detailed description, see [53].

Consider a multivariate random variable $\boldsymbol{V}$ with an underlying distribution $p_{\text{data}}(\boldsymbol{v})$. Intuitively, diffusion-based generative models iteratively transform samples from an initial noise distribution $p_{\mathscr{T}}$ into samples from the target data distribution p_{data} through a denoising operator. By convention, $\mathscr{T}$ is set to 1 as it is just nominal and does not correspond to the real time in the physical world. For clarity, we use τ and its capitalized version $\mathscr{T}$ to denote the diffusion times, which are different from the physical times. Additionally, as it will be clear from the explanation below, this initial distribution is a multivariate Gaussian distribution.

Noise is removed such that the samples follow a family of diffusion-time-dependent marginal distributions $p_\tau(\boldsymbol{v}_\tau;\sigma_\tau)$ for decreasing diffusion times τ and noise levels σ_τ [52]. The distributions are given by a forward blurring process that is described by the following stochastic differential equation (SDE) [53, 58]

$$d\boldsymbol{V}_\tau = f(\boldsymbol{V}_\tau,\tau)d\tau + g(\boldsymbol{V}_\tau,\tau)dW_\tau, \tag{S1}$$

with drift f, diffusion coefficient g, and the standard Wiener process W_τ. Following [58], we set

$$f(\boldsymbol{V}_\tau,\tau) = f(\tau)\boldsymbol{V}_\tau := \frac{\dot{s}_\tau}{s_\tau}\boldsymbol{V}_\tau, \qquad \text{and} \qquad g(\boldsymbol{V}_\tau,\tau) = g(\tau) := s_\tau\sqrt{2\dot{\sigma}_\tau\sigma_\tau}, \tag{S2}$$

where the overhead dot denotes the time derivative. Solving the SDE in (S1) forward in time with an initial condition $\boldsymbol{v}_0$ leads to the Gaussian perturbation kernel $p_\tau(\boldsymbol{V}_\tau|\boldsymbol{v}_0) = \mathscr{N}(s_\tau\boldsymbol{v}_0, s_\tau^2\sigma_\tau^2\mathbf{I})$. Integrating the kernel over the data distribution $p_0(\boldsymbol{v}_0) = p_{\text{data}}$, we obtain the marginal distribution $p_\tau(\boldsymbol{v}_\tau)$ at any τ. As such, one may prescribe the profiles of s_τ and σ_τ so that $p_0 = p_{\text{data}}$ (with $s_0 = 1, \sigma_0 = 0$), and more importantly

$$p_{\mathscr{T}}(\boldsymbol{V}_{\mathscr{T}}) \approx \mathscr{N}(0, s_{\mathscr{T}}^2\sigma_{\mathscr{T}}^2\mathbf{I}), \tag{S3}$$

i.e., the distribution at the (nominal) terminal time $\mathscr{T} = 1$ becomes indistinguishable from an isotropic, zero-mean Gaussian. To sample from p_{data}, we utilize the fact that the reverse-time SDE

$$d\boldsymbol{V}_\tau = \left[f(\tau)\boldsymbol{V}_\tau - g(\tau)^2\nabla_{\boldsymbol{V}_\tau}\log p_\tau(\boldsymbol{V}_\tau)\right]d\tau + g(\tau)dW_\tau \tag{S4}$$

has the same marginals as eq. (S1). Thus, by solving (S4) *backwards* using (S3) as the initial condition, we obtain samples from p_{data} at $\tau = 0$.

There are several approaches to learn the score function $\nabla_{\boldsymbol{V}_\tau}\log p_\tau(\boldsymbol{V}_\tau)$. The key step is to parameterize it with a neural network $s_\theta(\boldsymbol{v}_\tau,\sigma_\tau)$ such that

$$s_\theta(\boldsymbol{v}_\tau,\sigma_\tau) \approx \nabla_{\boldsymbol{v}_\tau}\log p_\tau(\boldsymbol{v}_\tau|\boldsymbol{v}_0) = -\frac{s_\tau\boldsymbol{v}_0 - \boldsymbol{v}_\tau}{\sigma_\tau^2} = z_\tau/\sigma_\tau \tag{S5}$$

for any $\boldsymbol{v} \sim p_\tau(\boldsymbol{V}_\tau|\boldsymbol{v}_0)$ and σ_τ. In other words, the *normalized* score network $\sigma_\tau s_\theta(\boldsymbol{v}_\tau,\sigma_\tau)$ identifies the noise z_τ injected into the data $\boldsymbol{v}_0$. The parameters θ of the score network are trained to reduce the L2 difference between them. Once this score network is learned, it is used to *denoise* a noised version of the desired data $\boldsymbol{v}_0$. We refer to this process as learning the *normalized score function*. In this work, we use the hyperparameters $f(\tau) = 0$ and $g(\tau) = 100^\tau$.

S2 Method Details

S2.1 Problem Setup and Main Idea

Here we provide a detailed description of the two generative tasks considered. Formally, let $p(\boldsymbol{v})$ denote an unknown distribution of the atmospheric state $\boldsymbol{V}$. In the task of **generative ensemble emulation**, we are

given a few examples or *seeds* sampled from $p(\boldsymbol{v})$ and our task is to generate more samples from the same distribution.

Given K samples $\mathscr{E}^K = (\boldsymbol{v}^1, \boldsymbol{v}^2, \cdots, \boldsymbol{v}^K)$ from $p(\boldsymbol{v})$, we construct an easy-to-sample *conditional* distribution

$$\hat{p}(\boldsymbol{v}) = p(\boldsymbol{v}; \mathscr{E}^K), \tag{S6}$$

which approximates $p(\boldsymbol{v})$. The conditional distribution $\hat{p}(\boldsymbol{v})$ needs to have two desiderata: it approximates well $p(\boldsymbol{v})$, and it is much less costly to sample than $p(\boldsymbol{v})$. Note that this problem extends the typical density estimation problem, where there is no conditioning, *i.e.*, $K = 0$.

In the task of **generative post-processing**, we construct an efficient sampler to approximate a mixture distribution. As another example, let $p'(\boldsymbol{v})$ be the reanalysis distribution of the atmospheric state corresponding to the forecast $p(\boldsymbol{v})$. We construct a conditional distribution $p(\boldsymbol{v}; \mathscr{E}^K)$ to approximate the following mixture of distributions:

$$p^{\text{MIX}}(\boldsymbol{v}) = \alpha p(\boldsymbol{v}) + (1-\alpha) p'(\boldsymbol{v}). \tag{S7}$$

In practice, the information about $p'(\boldsymbol{v})$ is given to the learning algorithm in the form of a representative K'-member ensemble $\mathscr{E}'^{K'}$. The mixture weight α is then given by

$$\alpha = \frac{M-K}{M-K+K'}. \tag{S8}$$

where $M > K$ is the number of samples we have drawn from $p(\boldsymbol{v})$, and $M-K$ is the number of samples from $p(\boldsymbol{v})$ we use to formulate the target (mixture) distribution, along with K' samples from $p'(\boldsymbol{v})$. In the following, we describe how to apply the technique of generative modeling introduced in S1 to these two tasks. We first mention how data is organized.

S2.2 Data for Training and Evaluation

Each ensemble $\mathscr{E}_{tl}$ is identified by the forecast initialization day t and the lead time l. Here, $t \in T^{\text{TRAIN}}$ indexes all the days in the training set and $l \in L$, where $L = \{1, 3, 6, 10, 13, 16\}$ days is the set of lead times we consider. We group ensembles of the same lead time l as a training dataset $\mathscr{D}_l^{\text{TRAIN}} = \{\mathscr{E}_{1l}, \mathscr{E}_{2l}, \cdots, \mathscr{E}_{T^{\text{TRAIN}}l}\}$. In generative ensemble emulation, $\mathscr{D}_l^{\text{TRAIN}}$ contains samples from GEFS-RF5 exclusively, since the target distribution is GEFS-Full. In generative post-processing, $\mathscr{D}_l^{\text{TRAIN}}$ contains samples from both GEFS-RF5 and ERA5-10, to captue the target distribution in eq. (S7).

Note that the K seeds can be included in the generated ensembles for evaluation. However, in this work, we decide to adopt a stricter evaluation protocol where the seeds are excluded from the generated ensembles; including them would mildly improve the quality of the generated ensembles, since the number of generated forecasts is far greater than K.

As a shorthand, we use $\boldsymbol{v}_t$ to denote all members in the ensemble forecast made at time t, and $\boldsymbol{v}_{tm}$ to denote the *mth* forecast at the same time.

S2.2.1 Generative Ensemble Emulation

In the ensemble emulation task we use K samples to construct a sampler of the conditional distribution $\hat{p}(\boldsymbol{v})$. To this end, for each lead time l and value of K, we learn a distinct conditional distribution model $\mathscr{M}_{lK}$. We choose $K = 1, 2, 3, 4$; and use $\mathscr{D}_l^{\text{TRAIN}}$ to learn $\mathscr{M}_{lK}$.

To train the conditional generation model $\mathscr{M}_{lK}$, we use as input data K randomly chosen members from each ensemble forecast $\boldsymbol{v}_t$ in $\mathscr{D}_l^{\text{TRAIN}}$, and another member $\boldsymbol{v}_{tm_0}$ from the remaining $(M-K)$ members as our target. This procedure augments the data in $\mathscr{D}_l^{\text{TRAIN}}$ by C_M^K-fold, where C_M^K is the number of K-combinations of the M members.

The learning goal is then to optimize the score network eq. (S5), so that the resulting sampler gives rise to:

$$p_{lK}(\boldsymbol{v}_{tm_0} | \boldsymbol{v}_{tm_1}, \boldsymbol{v}_{tm_2}, \cdots, \boldsymbol{v}_{tm_K}), \tag{S9}$$

where $m_1, m_2, \cdots, m_K$, and m_0 index the chosen ensemble members. Specifically, the score network is given by

$$s_\theta(\boldsymbol{v}_{tm,\tau}, \boldsymbol{v}_{tm_1}, \boldsymbol{v}_{tm_2}, \cdots, \boldsymbol{v}_{tm_K}, \mathbf{c}_t, \sigma_\tau) \tag{S10}$$

where $\boldsymbol{v}_{tm,\tau}$ is a perturbed version of $\boldsymbol{v}_{tm_0}$. We include the climatological mean $\mathbf{c}_t$ as an input to the score function eq. (S10), since the atmospheric state is non-stationary with respect to the time of the year.

S2.2.2 Generative Post-Processing

For the task of generative post-processing, the setup is similar except that we seek to approximate the mixture distribution of $p(\boldsymbol{v})$ and $p'(\boldsymbol{v})$ defined in eq. (S7). The training dataset $\mathscr{D}_l^{\text{TRAIN}}$ thus contains ensembles from two different distributions, $\boldsymbol{v}_t$ and $\boldsymbol{v}_t'$.

In this case, we train conditional distribution models $\mathscr{M}_{lKK'}$ for each triplet (l, K, K') using the same input as the conditional emulator $\mathscr{M}_{lK}$, *i.e.*, K out of M members of each ensemble $\boldsymbol{v}_t$. However, the target sample is now drawn randomly from either $p(\boldsymbol{v})$ or $p'(\boldsymbol{v})$. Consistent with eq. (S7), we draw a sample from the remaining $(M-K)$ members of ensemble $\boldsymbol{v}_t$ with probability $(M-K)/(M-K+K')$, or a member from $\boldsymbol{v}_t'$ with probability $K'/(M-K+K')$. Denoting the final selection as $\boldsymbol{x}_{t0}$, the score function becomes

$$s_\theta(\boldsymbol{x}_{t0,\tau}, \boldsymbol{v}_{tm_1}, \boldsymbol{v}_{tm_2}, \cdots, \boldsymbol{v}_{tm_K}, \mathbf{c}_t, \sigma_\tau) \tag{S11}$$

where $\boldsymbol{x}_{t0,\tau}$ is a perturbed version of $\boldsymbol{x}_{t0}$.

In this task, K' is a hyperparameter that controls the mixture distribution being approximated. We choose K' from 2, 3 or 4.

S2.3 Model Architecture

We employ an axial variant of the vision Transformer (ViT) [54] to model the normalized score functions $\sigma_\tau s_\theta(\cdot,\cdot)$ in eqns. (S10) and (S11), adapted to the particular characteristics of atmospheric modeling.

Each atmospheric state snapshot $\boldsymbol{v}$ is represented as a tensor v_{qp}, where q indexes the physical quantities and p the locations on the cubed sphere. In particular, the altitude levels of the same physical quantity are also indexed by q. The overall model operates on sequences of atmospheric state snapshots v_{sqp} where s indexes the sequence position. In the context of computer vision, this is similar to a video model, where p indexes the position in the image, q the "RGB" channels, and s the time.

The number of channels, which we use to model physical fields at different atmospheric levels, can be $q \sim \mathscr{O}(100)$ in our setting. For this reason, we resort to axial attention, applied to p, q, and s separately [55]. Each sequence dimension has its own learned position embedding instead of the usual fixed positional encodings.

S2.3.1 Spatial Embedding

We call a piece of data assigned to each grid point of the cubed sphere a slice. Regarding spatial attention, the $6 \times C \times C$ slices associated with the grid points indexed by p are partitioned into $6(C/P)^2$ square patches of size $P \times P$, where C^2 is the number of grid points per face in the cubed sphere. We employ $C = 48$ in our work, corresponding roughly to 2° resolution. Each patch is then flattened and linearly embedded into D dimensions with learned weights, leading to a tensor h_{sqld}, where $l = 0, \ldots 6(C/P)^2 - 1$ indexes the patches and d the in-patch dimension. Due to the nontrivial neighborhood structure of the cubed sphere, a shared learned positional encoding e_{ld}^{pos} is used for the l dimension. This is then fed into a transformer of T_L layers operating with l as the sequence dimension and d as the embedding dimension. The output is the slice embedding corresponding to each patch,

$$h_{sqld}^{\text{slice}} = T_L(h_{sqld} + e_{ld}^{\text{pos}}). \tag{S12}$$

For a schematic illustration, see Fig. S1.

S2.3.2 Atmospheric Field Embedding

The physical quantities have discrete semantic meanings, so we encode the positions in the q dimension using a sum of two shared learned embeddings e_{qd}^{field} and e_{qd}^{level}. We treat field labels like "mean sea-level pressure" and "eastward wind speed", and generalized levels like "surface", "2m", "500hPa", or "integrated" as strings. We then encode them with a categorical embedding. This is then fed into a transformer of T_F layers operating with q as the sequence dimension and d as the embedding dimension. The output is the snapshot embedding

$$h_{sqld}^{\text{snapshot}} = T_F(e_{qd}^{\text{field}} + e_{qd}^{\text{level}} + h_{sqld}^{\text{slice}}). \tag{S13}$$

For the schematic illustration, see Fig. S2.

S2.3.3 Sequence Embedding

The neural network operates on a sequence of snapshots. We tag each snapshot by adding two embeddings: a learned categorical embedding e^{type}_{sd} with values like "Denoise", "GEFS", "Climatology" for the type of the snapshot and a random Fourier embedding [59] e^{time}_{sd} for the relative physical time. In particular, the label "Denoise" denotes a snapshot for the score function input $\boldsymbol{v}$. For examples, GEFS ensemble members are labeled as type "GEFS" and the same relative physical time embedding, so to the sequence transformer, they are exchangeable as intended.

The score function of a diffusion model requires the diffusion time τ as an additional input, which we model by prepending to the sequence the token e^{τ}_{d}, derived from a random Fourier embedding of τ. The sequence and its embeddings are then fed into a transformer of T_S layers operating with s as the sequence dimension and d as the embedding dimension. The output embedding is

$$h^{\text{out}}_{sqld} = T_S(e^{\tau}_{d} \oplus_s (e^{\text{type}}_{sd} + e^{\text{time}}_{sd} + h^{\text{snapshot}}_{sqld})), \tag{S14}$$

where $\oplus_s$ means concatenation in the s dimension. This is illustrated in Fig. S3.

To obtain the score function $s_\theta(\boldsymbol{v}, \tau)$, we take the output token h^{out}_{1qld} matching the position of the denoise input and project it with learned weights and reshape back to a tensor v^{out}_{qp} as the final output.

S2.3.4 Hyperparameters

The most salient hyperparameters of this architecture are the patch size P, the model embedding dimension D, and the number of layers L_L, L_F, L_S of transformer stacks T_L, T_F, T_S. In our work, we use patch size $P = 12$ (number of patches 96), embedding dimension $D = 768$, and transformer layers $(T_L, T_F, T_S) = (6, 4, 6)$. The hidden layers in the feed-forward network have dimension $4D$ as usual. The model has 113,777,296 trainable parameters. These parameters are learned by training over randomly sampled days from the 20-year GEFS reforecast dataset [30], using a batch size 128 for 200,000 steps.

S3 Evaluation Metrics and More Results

Similar to how we organize the training data (cf. S2.2), we partition all evaluation data according to the lead time l, and generate one evaluation dataset $\mathscr{D}^{\text{EVAL}}_{l}$ for each training dataset $\mathscr{D}^{\text{TRAIN}}_{l}$. In the main text, we report results for ensembles generated by models $\mathscr{M}_{l2}$ and $\mathscr{M}_{l23}$, conditioned on $K = 2$ ensemble forecasts and in the case of generative post-processing blending the target distribution with $K = 3$ reanalysis samples.

S3.1 Evaluation Metrics

We use $\boldsymbol{v}$ (or $\boldsymbol{w}$) to denote ensemble forecasts, which are indexed by t (time), m (ensemble member ID), q (the atmospheric variable of interest), and p (the geolocation). We use $M_{\boldsymbol{v}}$ to denote the number of members in the ensemble, $\overline{\boldsymbol{v}}$ to denote the ensemble mean forecast, and $s_{\boldsymbol{v}}$ to denote the spread:

$$\overline{\boldsymbol{v}} = \frac{1}{M_{\boldsymbol{v}}} \sum_{m=1}^{M_{\boldsymbol{v}}} \boldsymbol{v}_m, \tag{S15}$$

$$s_{\boldsymbol{v}} = \sqrt{\frac{1}{M_{\boldsymbol{v}} - 1} \sum_{m} (\boldsymbol{v}_m - \overline{\boldsymbol{v}})^2}. \tag{S16}$$

We omit other indices (t, q and p) to avoid excessive subscripts, assuming the operations are applied element-wise to all of them.

S3.1.1 RMSE

The (spatial) root-mean-square-error (RMSE) between two (ensemble) means $\overline{\boldsymbol{v}}$ and $\overline{\boldsymbol{w}}$ is defined as

$$\text{RMSE}_t(\overline{\boldsymbol{v}}, \overline{\boldsymbol{u}}) = \sqrt{\frac{1}{P} \sum_{p=1}^{P} (\overline{\boldsymbol{v}}_{tp} - \overline{\boldsymbol{w}}_{tp})^2}, \tag{S17}$$

where p indexes all the geospatial locations. Again, this is computed element-wise with respect to every variable q. We summarize the RMSE along the temporal axis by computing the sample mean and variance,

$$\overline{\text{RMSE}}(\overline{\boldsymbol{v}},\overline{\boldsymbol{u}}) = \frac{1}{T}\sum_{t=1}^{T}\text{RMSE}_t(\overline{\boldsymbol{v}},\overline{\boldsymbol{u}}), \tag{S18}$$

$$s^2_{\text{RMSE}(\overline{\boldsymbol{v}},\overline{\boldsymbol{u}})} = \frac{1}{T-1}\sum_{t=1}^{T}\left(\text{RMSE}_t(\overline{\boldsymbol{v}},\overline{\boldsymbol{u}}) - \overline{\text{RMSE}}(\overline{\boldsymbol{v}},\overline{\boldsymbol{u}})\right)^2. \tag{S19}$$

Assuming the errors at different times are *i.i.d.*, the sample variance can be used to estimate the standard error of the mean estimate eq. (S18).

S3.1.2 Correlation Coefficients and ACC

The spatial correlation between two (ensemble) means $\overline{\boldsymbol{v}}$ and $\overline{\boldsymbol{w}}$ is defined as

$$\text{CORR}_t(\overline{\boldsymbol{v}},\overline{\boldsymbol{u}}) = \frac{\sum_p(\overline{\boldsymbol{v}}_{tp} - \overline{\overline{\boldsymbol{v}}_{tp}})(\overline{\boldsymbol{w}}_{tp} - \overline{\overline{\boldsymbol{w}}_{tp}})}{\sqrt{\sum_p(\overline{\boldsymbol{v}}_{tp} - \overline{\overline{\boldsymbol{v}}_{tp}})^2}\sqrt{\sum_p(\overline{\boldsymbol{w}}_{tp} - \overline{\overline{\boldsymbol{w}}_{tp}})^2}}, \tag{S20}$$

where $\overline{\cdot_{tp}}$ refers to averages in space.

The centered anomaly correlation coefficient (ACC) is defined as the spatial correlation between the climatological anomalies [35],

$$\text{ACC}_t(\overline{\boldsymbol{v}},\overline{\boldsymbol{u}}) = \frac{\sum_p(\overline{\boldsymbol{v}}'_{tp} - \overline{\overline{\boldsymbol{v}}'_{tp}})(\overline{\boldsymbol{w}}'_{tp} - \overline{\overline{\boldsymbol{w}}'_{tp}})}{\sqrt{\sum_p(\overline{\boldsymbol{v}}'_{tp} - \overline{\overline{\boldsymbol{v}}'_{tp}})^2}\sqrt{\sum_p(\overline{\boldsymbol{w}}'_{tp} - \overline{\overline{\boldsymbol{w}}'_{tp}})^2}}, \tag{S21}$$

where the anomalies are defined as the raw values minus the corresponding climatological mean $\boldsymbol{c}_{tp}$,

$$\overline{\boldsymbol{v}}'_{tp} = \overline{\boldsymbol{v}}_{tp} - \boldsymbol{c}_{tp}, \quad \overline{\boldsymbol{w}}'_{tp} = \overline{\boldsymbol{w}}_{tp} - \boldsymbol{c}_{tp}. \tag{S22}$$

Both estimates eq. (S20) and (S21) are then averaged in time over the evaluation set.

S3.1.3 CRPS

The continuous ranked probability score (CRPS) [60] between an ensemble and ERA5 is given by

$$\text{CRPS}(\boldsymbol{v},\text{ERA5}) = \frac{1}{M_{\boldsymbol{v}}}\sum_{m=1}^{M_{\boldsymbol{v}}}|\boldsymbol{v}_m - \text{ERA5}| - \frac{1}{2M_{\boldsymbol{v}}^2}\sum_{m=1}^{M_{\boldsymbol{v}}}\sum_{m'=1}^{M_{\boldsymbol{v}}}|\boldsymbol{v}_m - \boldsymbol{v}_{m'}|. \tag{S23}$$

This is computed for all time and geo-spatial locations, and then averaged.

We use the traditional CRPS instead of the ensemble-adjusted CRPS* [6]. The latter examines properties of ensembles in the theoretical asymptotic limit where their sizes are infinite. Since our approach aims at *augmenting* the physics-based ensembles, the definition eq. (S23) is more operationally relevant; even perfect emulation would obviously not improve the CRPS* score. Indeed, our model SEEDS-GEE can be seen as a practical way to bridge the skill gap between a small ensemble and the asymptotic limit.

S3.1.4 Rank Histogram, Unreliability, and Reliability Diagram

We assess the reliability of the ensemble forecasts in the main text in terms of their rank histograms [36]. An ideal ensemble forecast should have a flat rank histogram, so deviations from a flat rank histogram indicate unreliability.

We aggregate rank histograms for all n_{testing} dates in the evaluation set to obtain the average rank histogram $\{s_i\}_{i=0}^{M_{\boldsymbol{v}}}$, where s_i is the number of times the label has rank i and $M_{\boldsymbol{v}}$ is the ensemble size. Following Candille

and Talagrand [38], the unreliability Δ of the ensemble is defined as the squared distance of this histogram to a flat histogram

$$\Delta = \sum_{i=0}^{M_{\boldsymbol{v}}} \left(s_i - \frac{n_{\text{testing}}}{1+M_{\boldsymbol{v}}} \right)^2 . \tag{S24}$$

This metric has the expectation $\Delta_0 = n_{\text{testing}} \frac{M_{\boldsymbol{v}}}{1+M_{\boldsymbol{v}}}$ for a perfectly reliable forecast. The unreliability metric δ is defined as

$$\delta = \frac{\Delta}{\Delta_0}, \tag{S25}$$

which has the advantage that it can be used to compare ensembles of different sizes. Once δ is computed for each location, a global δ value is obtained by averaging over all locations. We further assess reliability through the reliability diagram in S3.2.3. We obtain the diagrams following the CORP approach of [61].

S3.1.5 Brier Score for Extreme Event Classification

To measure how well the ensembles can predict extreme events, we first apply a binarization criterion $b : \mathbb{R} \rightarrow \{0, 1\}$ (such as "if T2M is 2σ away from its mean, issue an extreme heat warning."), and verify against the classification of the same event by the ERA5 HRES reanalysis,

$$b_{tqp}^{\text{ERA5}} = b(\text{ERA5}_{tqp}), \tag{S26}$$

by converting the ensemble forecast into a probabilistic (Bernoulli) prediction

$$b_{tqp}^{\boldsymbol{v}} = \frac{1}{M_{\boldsymbol{v}}} \sum_{m=1}^{M_{\boldsymbol{v}}} b(\boldsymbol{v}_{tmqp}). \tag{S27}$$

We can evaluate how well the probabilistic predictions align with the discrete labels by evaluating the Brier score [62]

$$\text{BRIER}^b(\boldsymbol{v}, \text{ERA5})_{tq} = \frac{1}{P} \sum_{p=1}^{P} (b_{tqp}^{\text{ERA5}} - b_{tqp}^{\boldsymbol{v}})^2. \tag{S28}$$

Alternatively, the probabilistic predictions can be evaluated by their cross-entropy, also known as the logarithmic loss [63]

$$\text{LOGLOSS}^b(\boldsymbol{v}, \text{ERA5})_{tq} = -\frac{1}{P} \sum_{p=1}^{P} b_{tqp}^{\boldsymbol{v}} \ln b_{tqp}^{\text{ERA5}} + (1 - b_{tqp}^{\boldsymbol{v}}) \ln(1 - b_{tqp}^{\text{ERA5}}). \tag{S29}$$

It is possible for all ensemble members to predict the wrong result (especially for small ensembles), leading to the $\text{LOGLOSS}^b = -\infty$ due to $\ln(0)$. In practice, a small number $\varepsilon = 10^{-7}$ is added to both logarithms since no forecast is truly impossible [63]. The value of the metric is thus affected by the choice of ε, a known inconvenience of this otherwise strictly proper metric.

S3.1.6 GEFS Model Climatological Spread

The baseline GEFS-Climatology spread in Fig. 8 is computed from the training dataset, which contains 20 years of GEFS reforecasts [30]. The climatological spread is computed for each lead time independently. For a fixed lead time, the point-wise spread of the 5-member ensemble is computed for each day in the dataset. Then the model climatological spread for that lead time is defined as the day-of-year average over the 20 years.

Note that we slightly abuse standard notation, since this spread accounts not only for the internal variability of the model, but also for forecast uncertainty [64]. The spread computed this way serves as a baseline that is independent of the particular initial conditions at the time of the forecast.

S3.1.7 Spatial coherence and energy spectra

An important aspect of realistic weather forecasts is spatial coherence, which can be characterized by slowly-varying spatial decorrelation length scales, and the spatial alignment of features in different fields. For example, a trough in mean sea-level pressure may correspond to a local minimum in midtropospheric geopotential with similar spatial extent.

Spatial structure of weather forecasts can be quantitatively assessed by comparing the energy distribution across length scales to the distribution observed in the reanalysis. This distribution is known as the energy spectrum, which for atmospheric fields displays a characteristic energy cascade with important implications for the redistribution of energy across scales. We compute the energy spectrum of a field on a global latitude-longitude grid by applying the 2D Discrete Fourier Transform, and computing the total energy as a function of length scale. In the main text we provide the mean energy spectrum for each field by averaging over 1 month of time. For ensemble forecasts, the energy spectrum is estimated for each member, and the ensemble mean and standard deviation of the spectra are provided.

S3.2 Detailed and Additional Results

Unless noted, the results reported here are obtained from $N = 512$ generated ensembles from our models, with $K = 2$ seeding forecasts and $K' = 3$ for SEEDS-GPP.

S3.2.1 RMSE, ACC, CRPS, Reliability for All Fields

We report values for RMSE (Fig. S4), ACC (Fig. S5), CRPS (Fig. S6), and δ (Fig. S7) for all 8 modeled fields in Table 2. The results are consistent with those reported in the main text: SEEDS ensembles attain similar or better skill than the physics-based ensembles, with SEEDS-GPP performing better than SEEDS-GEE for variables that are biased in GEFS-Full. In addition, SEEDS-GPP is in general more reliable than GEFS-Full and SEEDS-GEE, particularly within the first forecast week.

S3.2.2 Brier score of all fields at various thresholds

In Fig. S8, we report the Brier score at different thresholds: $\pm 3\sigma, \pm 2\sigma,$ and $\pm 1\sigma$, respectively. For all thresholds, SEEDS-GPP provides the most accurate extreme forecast ensembles among all others, for most lead times and variables. Moreover, the generative ensembles from SEEDS-GPP and SEEDS-GEE perform particularly well for longer lead times and more extreme prediction tasks. For the most extreme thresholds considered ($\pm 3\sigma$), the generative ensembles in general outperform GEFS-Full.

S3.2.3 Reliability diagram

Fig. S9 illustrates the CORP reliability diagrams [61] for mean sea level pressure, 2m temperature, and 850 hPa meridional wind forecasts in the upper and lower terciles of their climatological distribution. Results for the upper and lower 5-percentiles are also shown. These demonstrate that SEEDS-GPP is much more reliable at predicting the tails of the distribution than GEFS-Full.

S3.3 Effect of N, K and K'

S3.3.1 Generative ensemble emulation with varying N for 7-day lead time with $K = 2$

Fig. S10 shows the effect of N, the size of the generated ensemble, on the skill of SEEDS-GEE forecasts. Some metrics, such as Brier scores for 3σ, are omitted as they convey similar information.

For many metrics, increasing N offers diminishing marginal gains beyond $N = 256$. Metrics which are sensitive to the sampling coverage and the forecast distribution tails, such as the unreliability δ and the Brier score at -3σ, still improve with larger $N > 256$.

S3.3.2 Generative ensemble emulation with varying K for 7-day lead time

Fig. S11 studies the effect of K, the number of seeds, on the skill of SEEDS-GEE forecasts. We find that the ensemble forecast skill of SEEDS-GEE is comparable to physics-based *full* ensembles, when conditioned on $K \geq 2$ seeds from GEFS. Note that SEEDS-GEE is a drastically better forecast system than physics-based ensembles formed by the conditioning members (GEFS-2).

For all values of K, the affordability of large ensemble generation warranted by SEEDS leads to very significant improvements in extreme event classification skill with respect to the conditioning ensemble. It is also shown that the generative emulation approach is also feasible when $K = 1$. However, the skill of the generative ensemble is closer to physics-based ensembles with just a few members (GEFS-2) than to GEFS-Full. These studies suggest that $K = 2$ could be a good compromise between generative ensemble performance and computational savings.

S3.3.3 Generative post-processing with varying K' for 7-day lead time with $K = 2$

Fig. S12 studies the effect of K', which defines the mixture weight α, on the skill of SEEDS-GPP forecasts. Since the training data has ensemble size 5, the number of available GEFS training labels is $M - K = 3$. We see no substantial forecast skill gains blending in beyond 4 reanalyses members (around α =40% mixing ratio). SEEDS-GPP models trained with mixing ratios in the range $\alpha \in (20, 50)$ % have similar skill and spread correlation with GEFS-Full.

In the case where only the ERA5 reanalysis was used as the label to train SEEDS-GPP, so $M - K = 0$ and $\alpha =$ 0%, both the spread correlation and the skill of the generated ensemble deteriorate. This validates targeting a mixture distribution as an optimal strategy to reduce forecast biases and maintain ensemble spread. In the main text, we report results for $K' = 3$ ($\alpha = 50\%$).

S3.3.4 Case Study: Visualization of Generated Ensembles

Ensemble forecasts are samples drawn from multivariate high-dimensional distributions. Making comparisons among these distributions using a limited number of samples is a challenging statistical task. In this case study, we examine several ensembles by focusing on two fields at a single spatial location to reduce the problem dimensionality.

Figures S13 and S14 visualize the joint distributions of temperature at 2 meters and total column water vapour at the grid point near Lisbon during the extreme heat event on 2022/07/14 at 1:00 local time. We used the 7-day forecasts made on 2022/07/07. For each plot, we generate 16,384-member ensembles, represented by the orange dots using different seeds from the 31-member GEFS ensemble. The density level sets are computed by fitting a kernel density estimator to the ensemble forecasts. The observed weather event is denoted by the star.

In Figure S13, we compare SEEDS-GEE and SEEDS-GPP generated samples conditioned on 6 different two seeding forecasts from the 31-member GEFS ensemble. The plots are arranged in 2 pairs of rows (4 rows in total) such that all the odd rows are from SEEDS-GEE and even rows are from SEEDS-GPP. For each row pair and column, the same seeds are used. Given the same seeds, the SEEDS-GPP distribution is correctly more biased towards warmer temperatures while maintaining the distribution shape and spread compared to SEEDS-GEE.

In Figure S14 we compare SEEDS-GEE based on 2 seeds with SEEDS-GEE based on 4 seeds. We first generate samples conditioned on 4 randomly sampled seeding forecasts from the 31-member GEFS ensemble. Then we generate samples conditioned on 2 randomly selected seeding forecasts from the 4 chosen in the first round. The plots show 6 such pairs of samples, arranged in 2 pairs of rows (4 rows in total) such that all odd rows use 2 seeds and even rows use 4 seeds. For each row pair and column, the 4 seeds below always contain the 2 seeds above. We observe that the distribution of the generated samples conditioned on 4 seeds are more robust with respect to seed sampling. More seeds also lead to better coverage of the extreme heat event by the generated envelopes, at the expense of a greater total forecast cost.

S3.4 Comparison with quantile-mapped GEFS-Full

Figure S15 compares 7-day forecasts from the SEEDS-GPP ensemble against a post-processed version of GEFS-Full quantile-mapped to the ERA5 climatology. Quantile mapping [46] is performed based on the reference 20 year period for which data from the 5-member GEFS reforecast ensemble is available. The cumulative distribution functions (CDFs) from GEFS and ERA5 are constructed using point-wise isotonic regression [61]. Then, the GEFS CDF for each variable is mapped to the ERA5 CDF to correct systematic biases of the forecast system [65]. As in the main text, SEEDS-GPP samples are generated conditioned on 2 random seeds, and have a mixing ratio of $\alpha = 50\%$.

The post-processed GEFS-Full ensemble has better forecast skill than the original ensemble at the tails, as shown by the lower Brier score for -3σ events. RMSE and ACC scores are also improved for 2-meter temperature, due to the reduction in quantile-dependent biases. For less biased variables, quantile mapping can lead to higher RMSE and CRPS [65], as shown for the mean sea level pressure. In comparison, SEEDS-GPP shows lower CRPS and -3σ Brier scores than the quantile-mapped GEFS, proving its added value as a debiased probabilistic forecasting system. The deterministic scores of both models are roughly similar, with SEEDS-GPP having in general lower RMSE, but also achieving a lower ACC than the post-processed GEFS-Full. Scores for the conditioning and non-debiased ensembles are also shown for reference.

S4 Scaling of computational cost with resolution

The current models have a throughput of 256 members per 3 minutes, at 2° resolution, on a single Google Cloud TPUv3-32 instance (https://cloud.google.com/tpu). Assuming a linear scaling of computational cost with the number of grid points, then we would expect that it would take about 2 hours to generate a global ensemble of 256 members at 10 km resolution, using the same computational hardware used for generating the results in the paper. Nevertheless, this computation is easily parallelizable by procuring more cost-effective cloud-based accelerators.

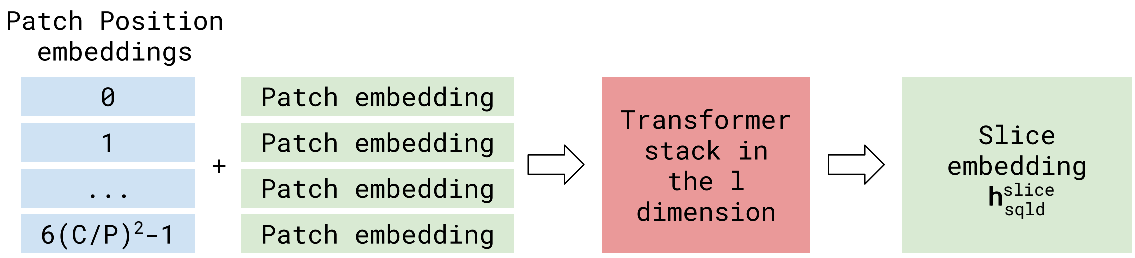

Fig. S1. The l-dimension transformer stack of the axial transformer. The input is a combination of the embeddings of the spatial patches (data from one field at a time associated with cubed sphere grid points) and their corresponding patch position embeddings. The output is a slice embedding.

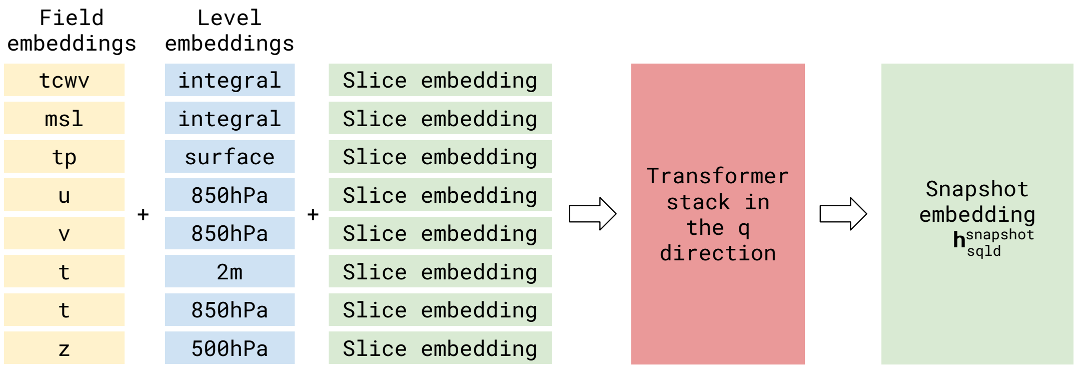

Fig. S2. The q-dimension transformer stack of the axial transformer. The input is a combination of the slice embeddings and their corresponding field names and levels embeddings. The output is a snapshot embedding.

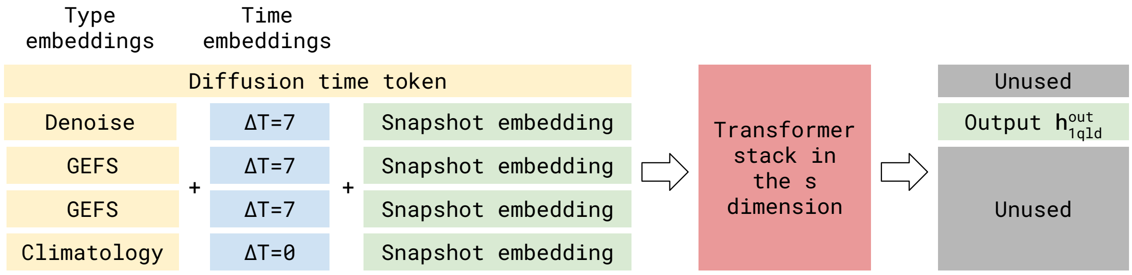

Fig. S3. The *s*-dimension transformer stack of the axial transformer. The input is a combination of the snapshot embeddings and their corresponding type and time embeddings, prepended with the diffusion time embeddings. The output embeddings corresponding to the first snapshot is further projected back to the required shape as the final output.

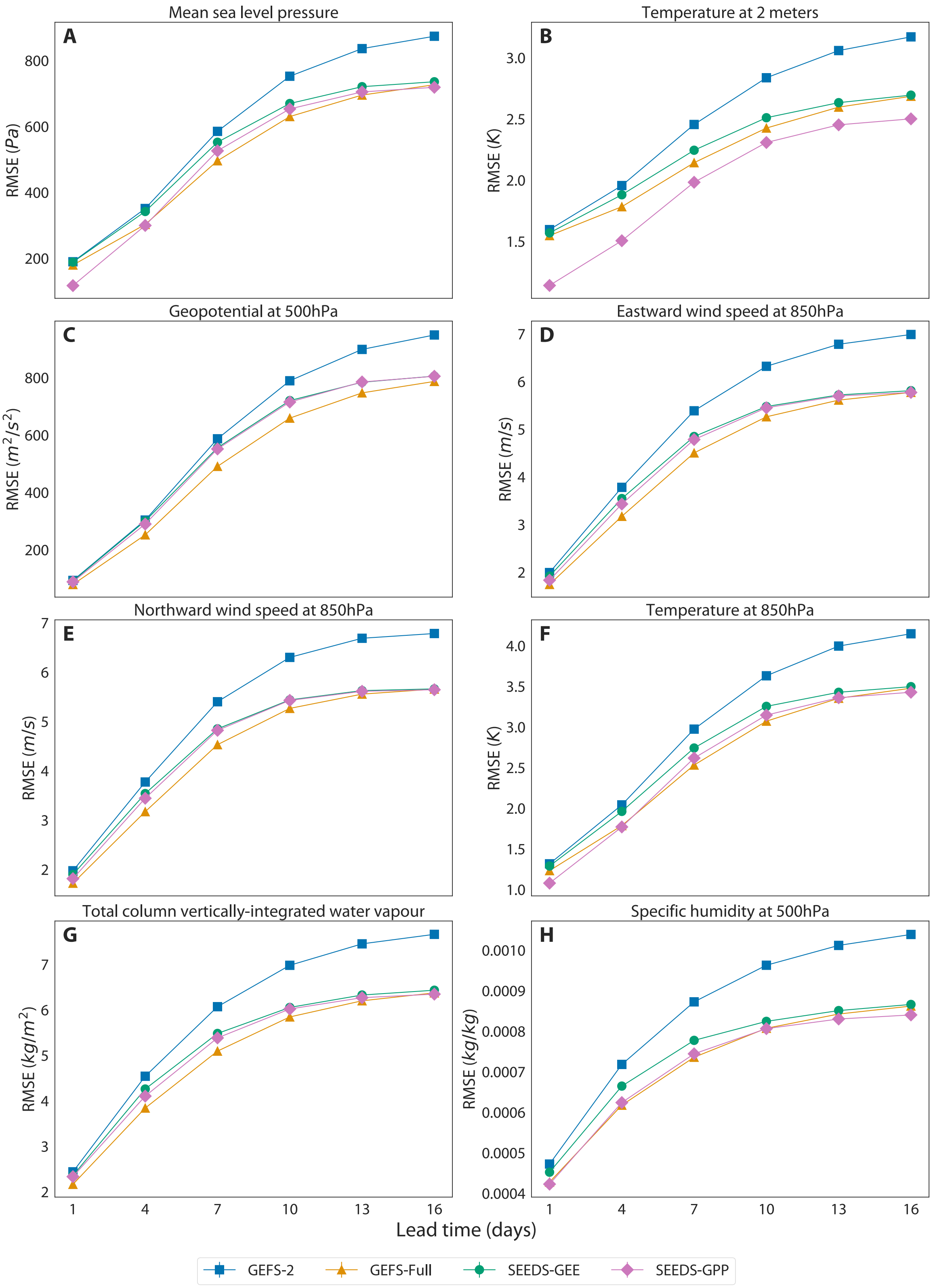

Fig. S4. Comparison of the RMSE of the ensemble means with ERA5 as the label. Global data from ERA5 for the entire 2022 is the ground truth. Both SEEDS-GEE and SEEDS-GPP have 512 samples generated from 2 random seeds GEFS-2 from the GEFS-Full ensemble. Panels (A–H) show the results for all the 8 fields modeled.

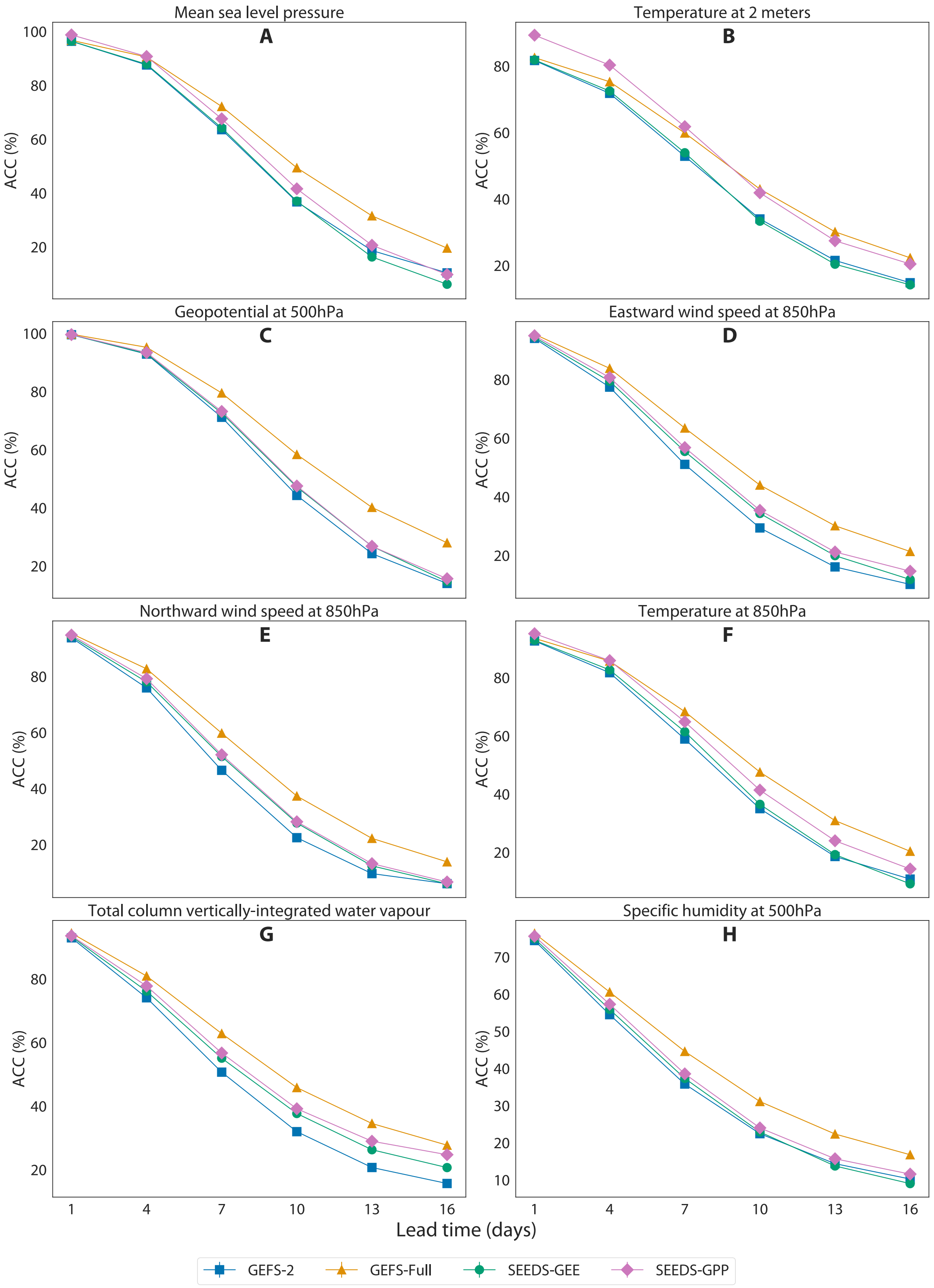

Fig. S5. Comparison of the ACC of the ensemble means with ERA5 as the label. Global data from ERA5 for the entire 2022 is the ground truth. Both SEEDS-GEE and SEEDS-GPP have 512 samples generated from 2 random seeds GEFS-2 from the GEFS-Full ensemble. Panels (A–H) show the results for all the 8 fields modeled.

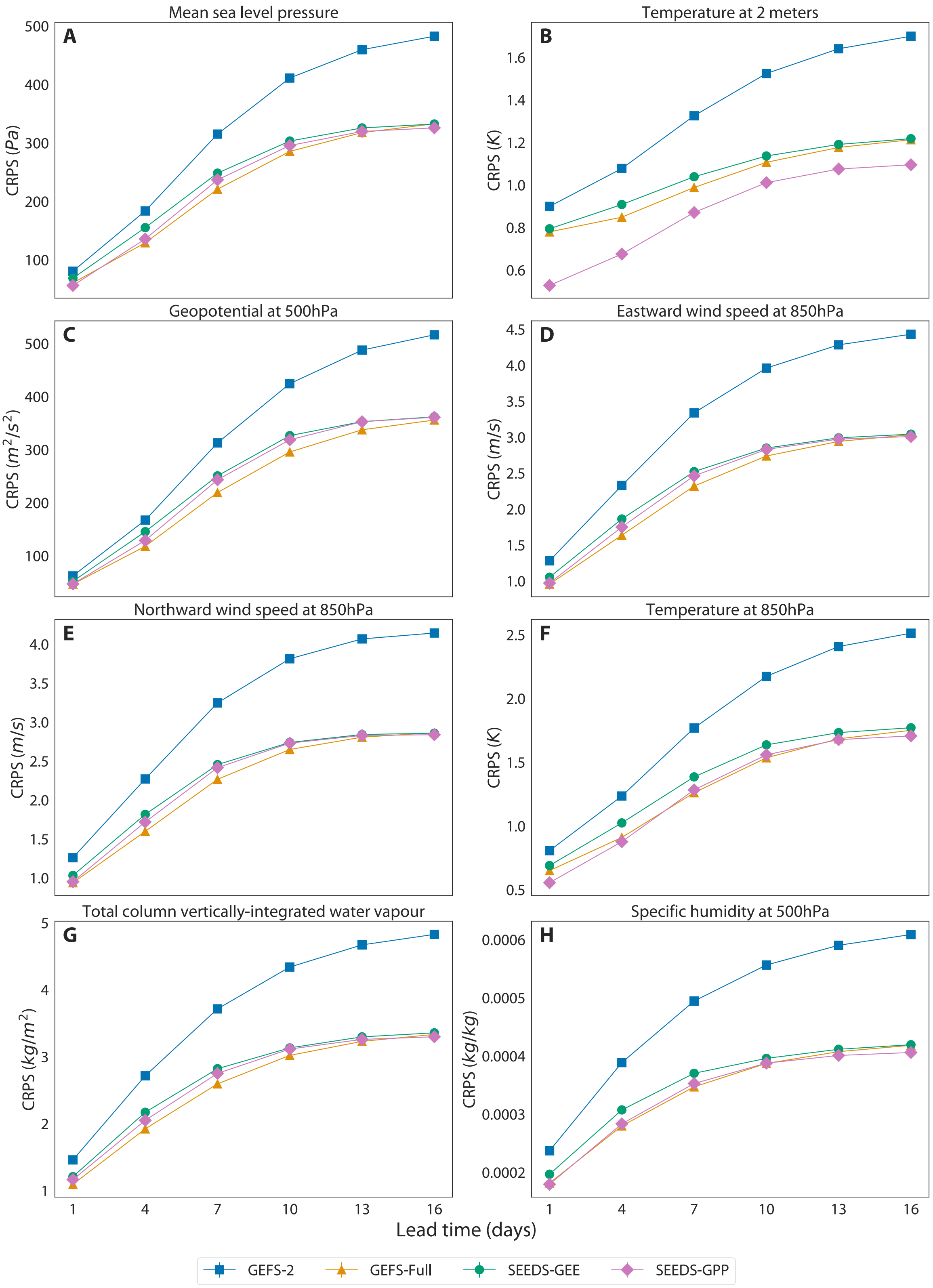

Fig. S6. Comparison of the CRPS of the ensembles with ERA5 as the label. Global data from ERA5 for the entire 2022 is the ground truth. Both SEEDS-GEE and SEEDS-GPP have 512 samples generated from 2 random seeds GEFS-2 from the GEFS-Full ensemble. Panels (A–H) show the results for all the 8 fields modeled.

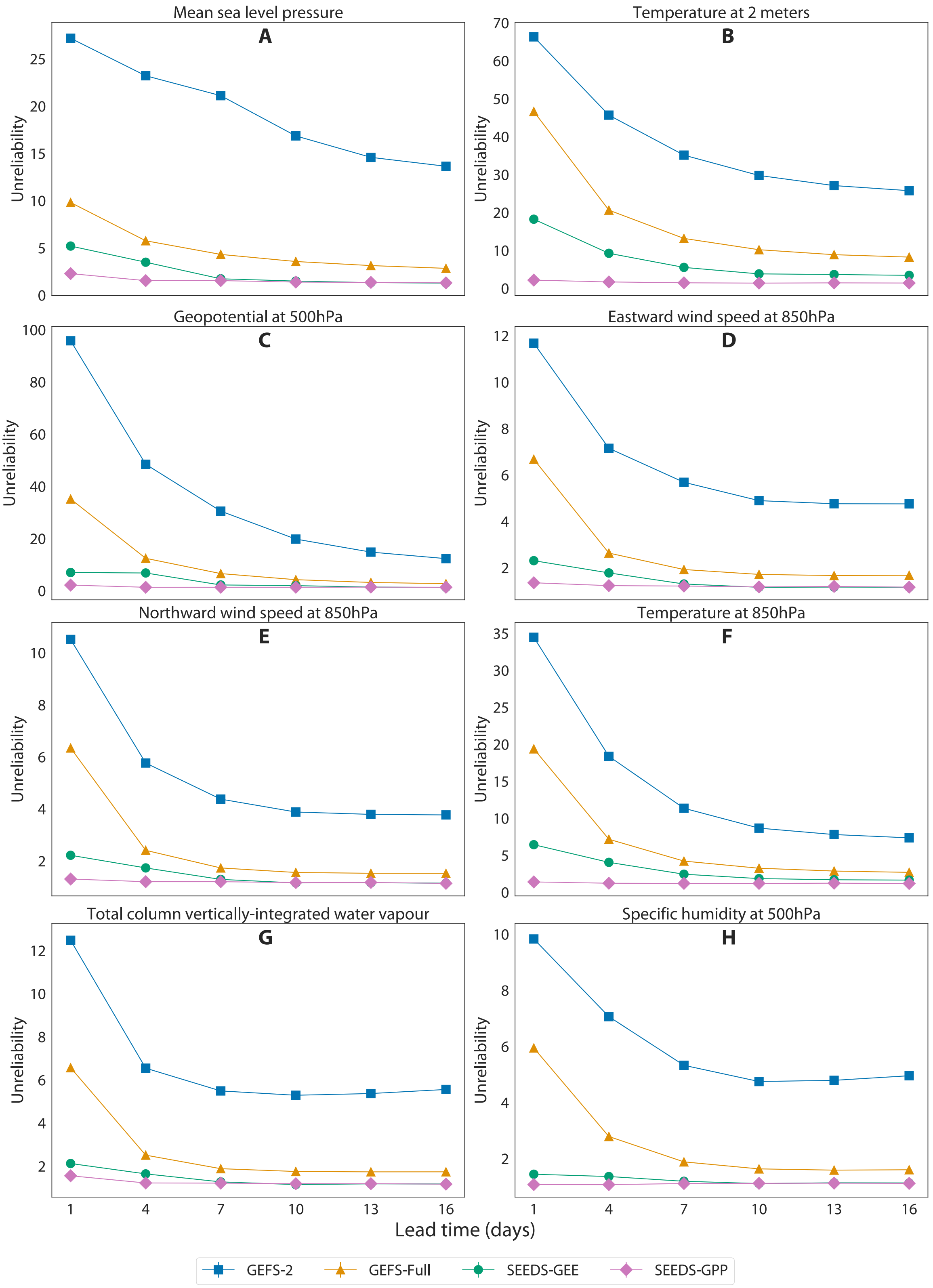

Fig. S7. Comparison of the unreliability δ of the ensembles with ERA5 as the label. Global data from ERA5 for the entire 2022 is the ground truth. Both SEEDS-GEE and SEEDS-GPP have 512 samples generated from 2 random seeds GEFS-2 from the GEFS-Full ensemble. Panels (A–H) show the results for all the 8 fields modeled.

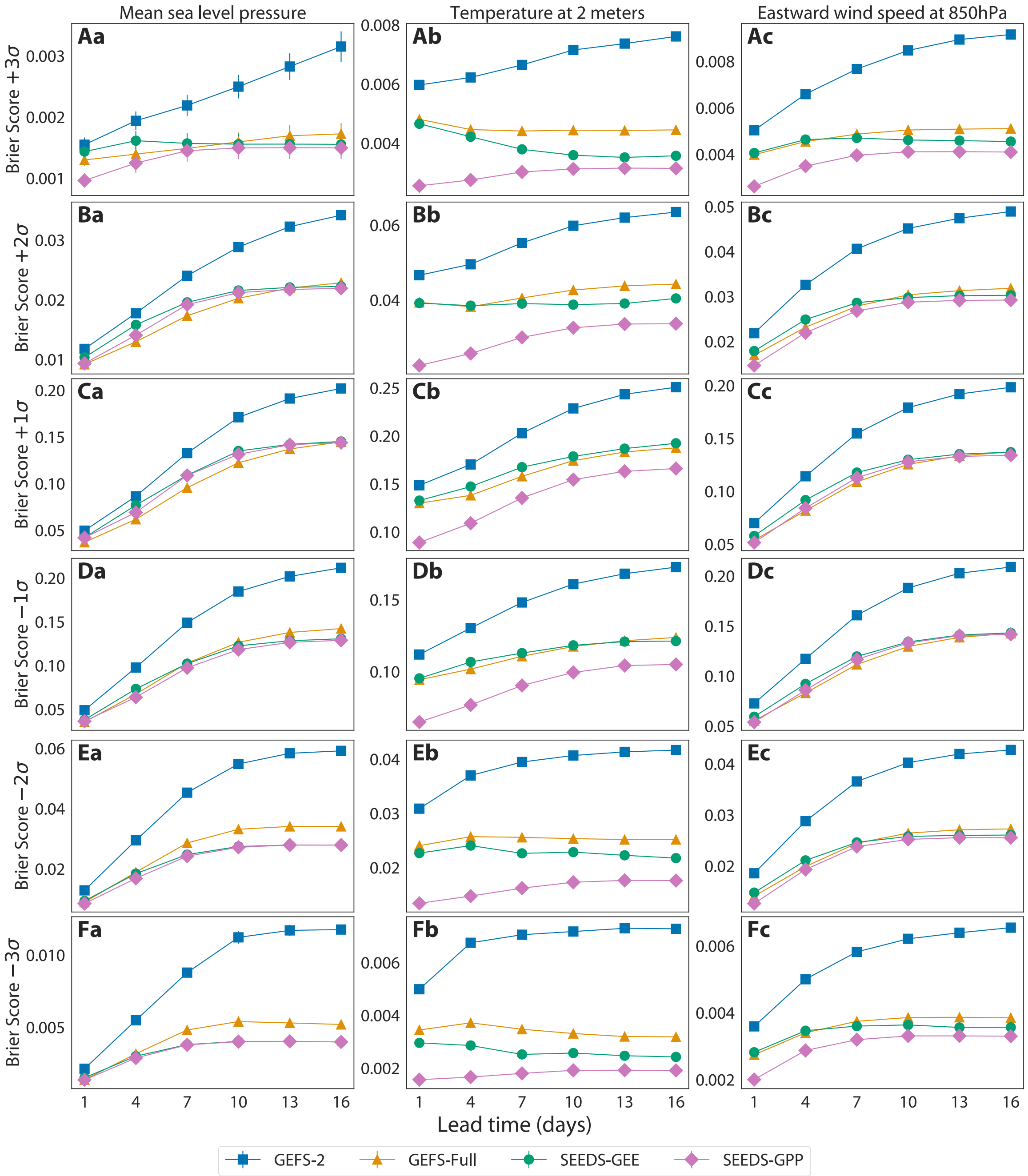

Fig. S8. Brier scores at different levels with ERA5 as the label. Global data from ERA5 for the entire 2022 is the ground truth. Both SEEDS-GEE and SEEDS-GPP have 512 samples generated from 2 random seeds GEFS-2 from the GEFS-Full ensemble. Panel rows (A–F) show the results at levels from -3σ to $+3\sigma$ and columns (a–c) show the results for 3 fields.

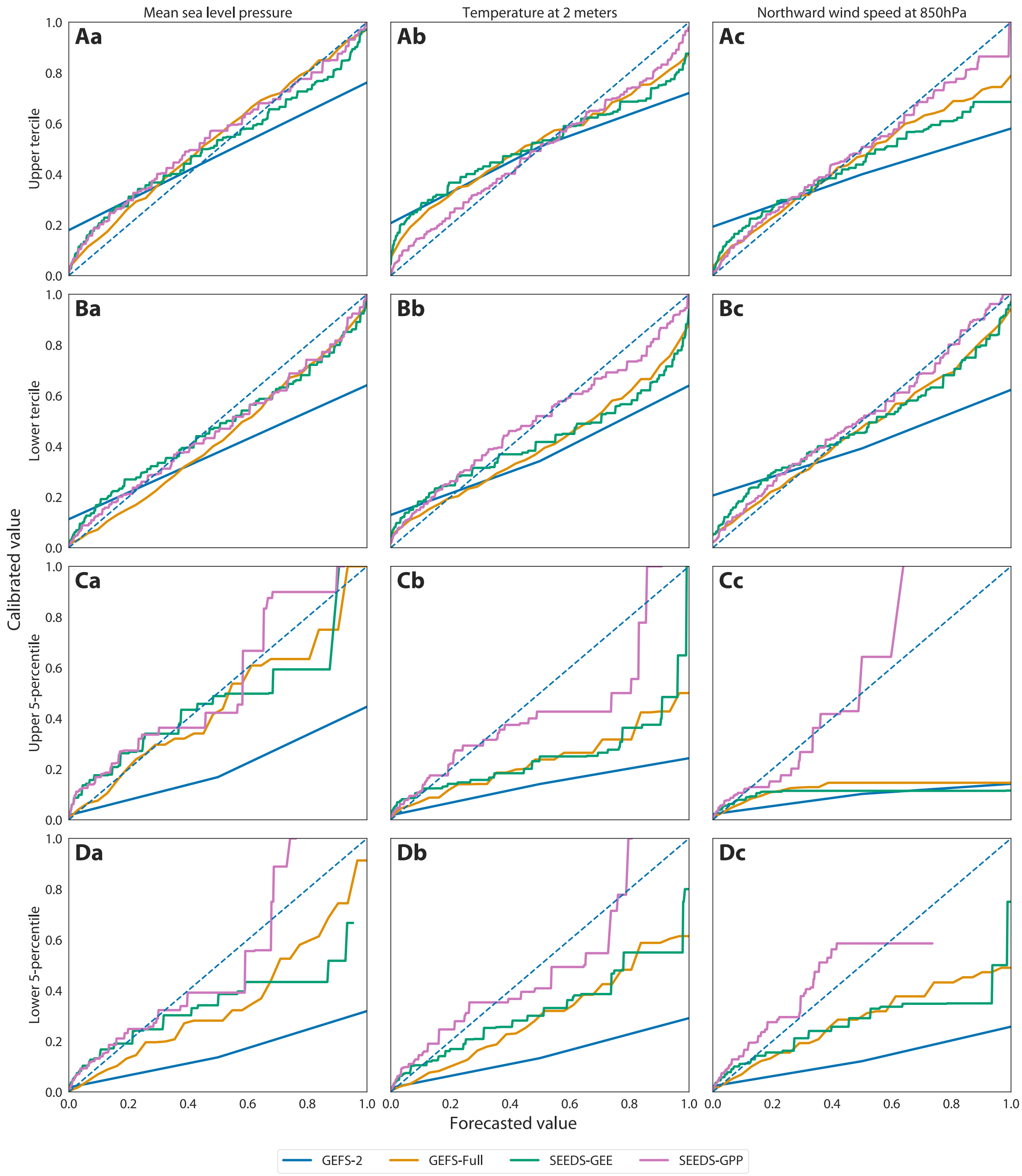

Fig. S9. Reliability diagrams of the physical and generative forecast ensembles with ERA5 as the label. These are computed for the rectangular region 66W–125W and 24N–50N, roughly containing the contiguous United States, for 7-day lead time forecasts for the entire year 2022. SEEDS-GEE and SEEDS-GPP samples are generated using 2 random seeds from GEFS-Full. Panel rows (A) and (B) show the reliability diagrams at $\pm 0.43\sigma$ levels with respect to climatology, representing approximately the upper and lower terciles, while (C) and (D) focus on the $\pm 1.64\sigma$ tails, or approximately the 5-percentile levels. Panel columns are for (a) mean sea level pressure, (b) temperature at 2 meters, and (c) northward wind speed at 850hPa.

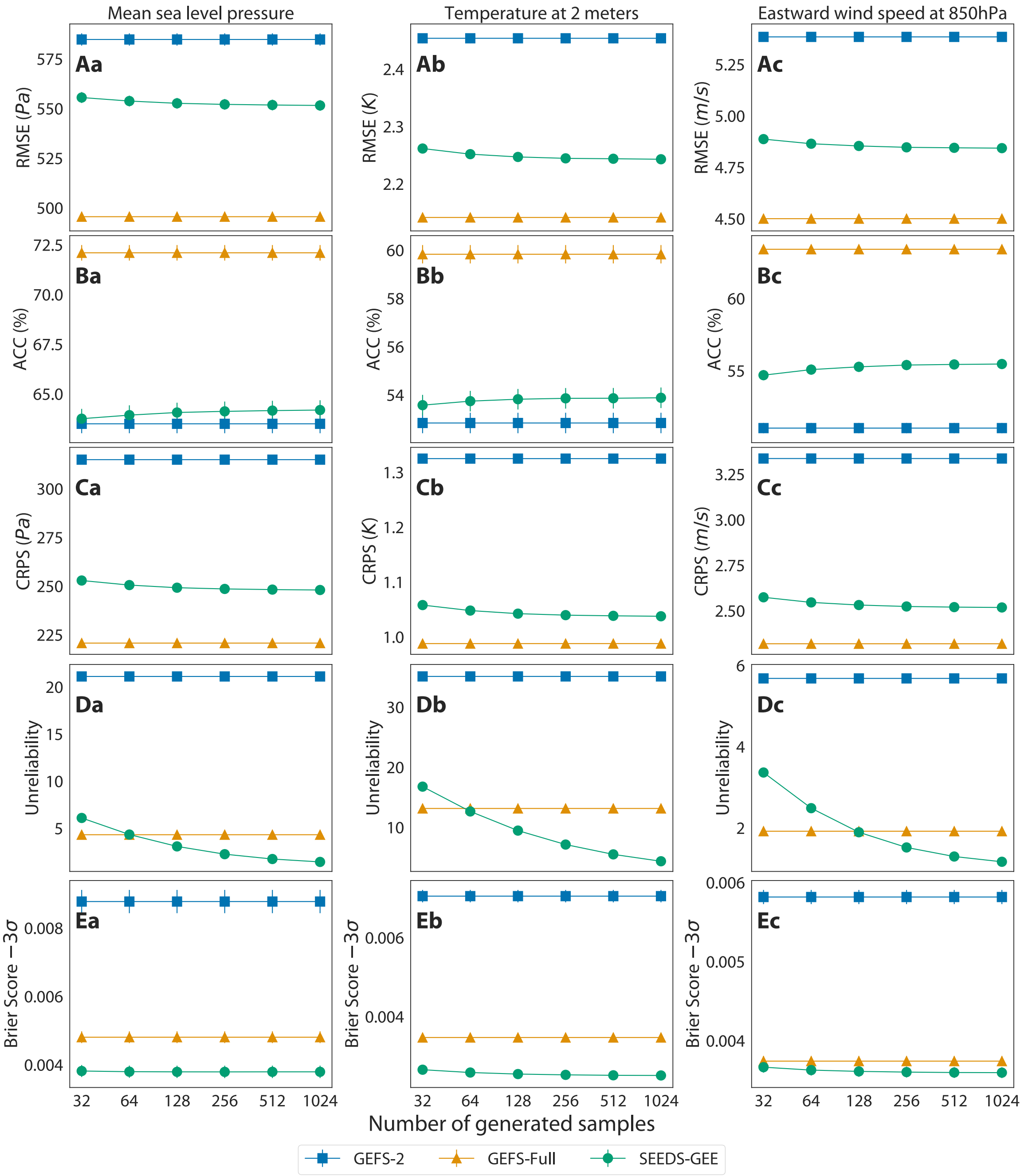

Fig. S10. The effect of generated sample size N on skill metrics. Panel rows (A–E) are for metrics RMSE, ACC, CRPS, unreliability index, and Brier Score at -3σ, for 7-day forecasts issued in 2022. Columns are for fields (a) mean sea level pressure, (b) temperature at 2 meters, and (c) eastward wind speed at 850hPa. SEEDS-GEE samples are generated using the same 2 random seeds from GEFS-Full at increasing sample sizes. ERA5 is the label for evaluation.

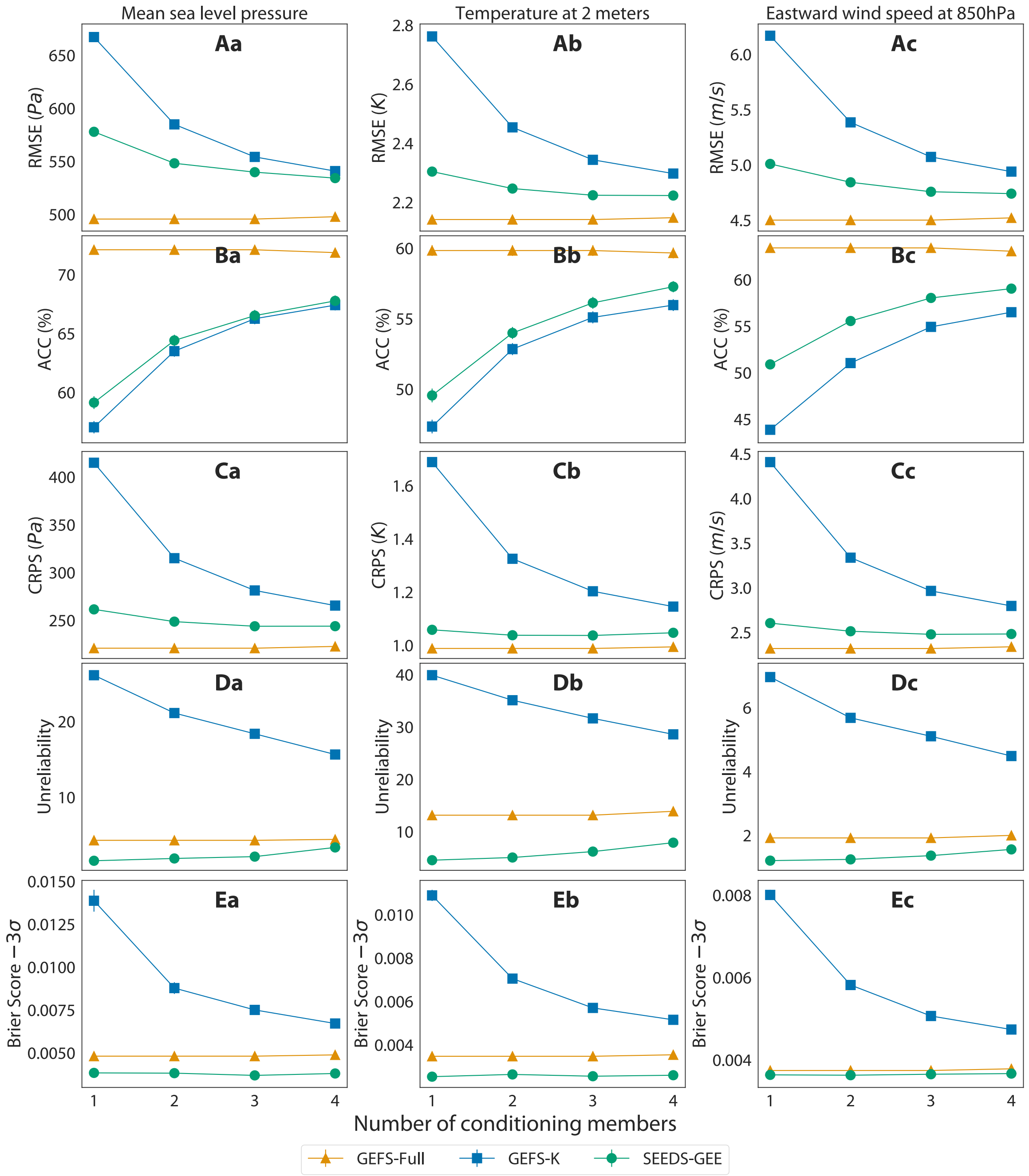

Fig. S11. The effect of the number of seeds K on skill metrics. Panel rows (A–E) are for metrics RMSE, ACC, CRPS, unreliability index, and Brier Score at -3σ, for 7-day forecasts issued in 2022. Columns are for fields (a) mean sea level pressure, (b) temperature at 2 meters, and (c) eastward wind speed at 850hPa. In all panels, 512 SEEDS-GEE samples are generated using the K random seeds from GEFS-Full, and ERA5 is the label for evaluation.

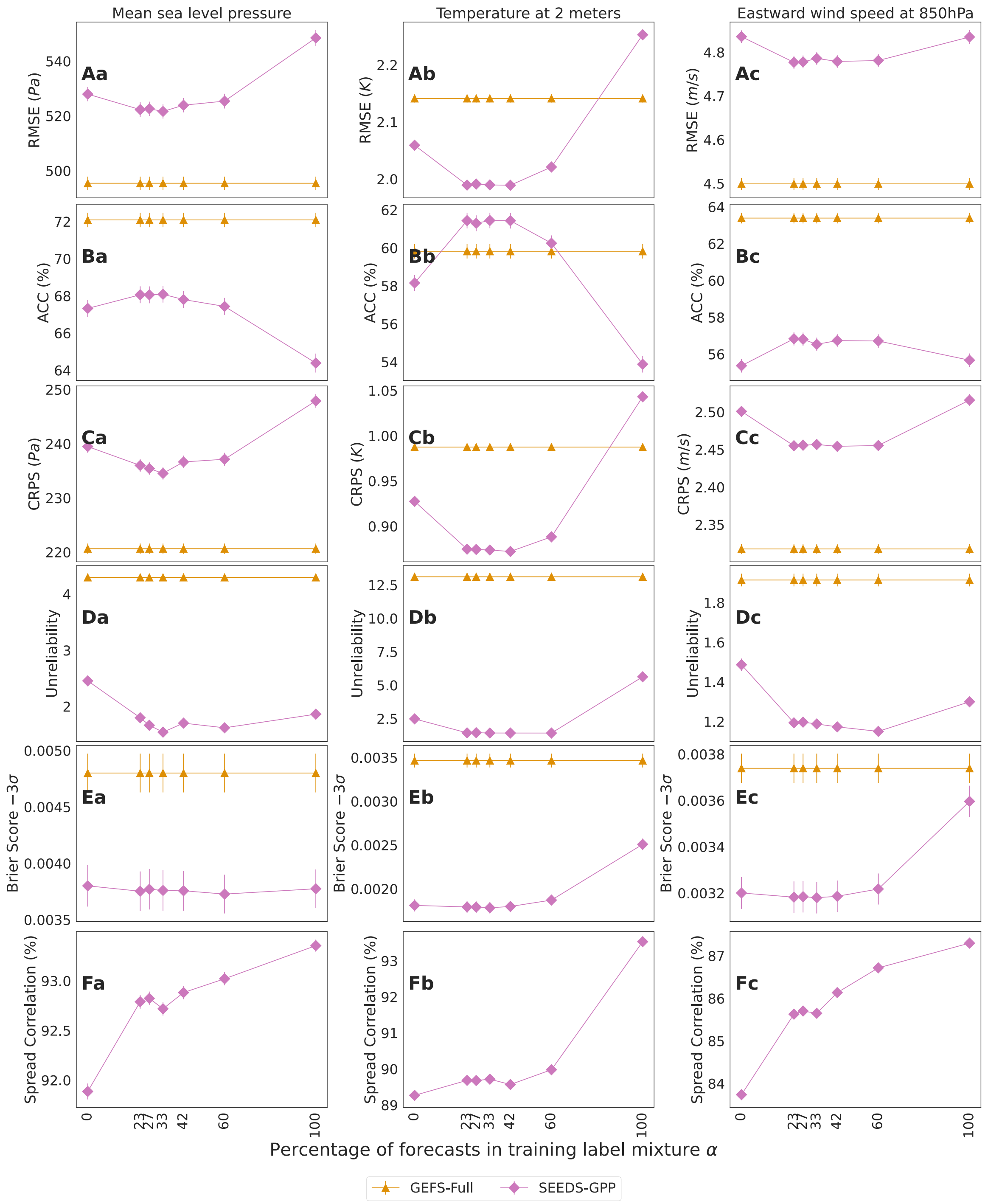

Fig. S12. The effect of mixing ratio α on SEEDS-GPP metrics. The lead time is 7 days. 512 SEEDS-GPP samples are generated using the same 2 random seeds from GEFS-Full, and ERA5 for the entire 2022 is the label for evaluation. The nonzero mixing ratios are $\alpha = 3/(3+K')$ for the number of reanalyses $K' = 2,4,6,8,10$, mixed with 3 forecasts (the other 2 members of GEFS-RF5 are the seeds). The case $\alpha = 0$ is special and is trained with only ERA5 as the label. Panel rows (A–E) are for metrics RMSE, ACC, CRPS, Unreliability index, Brier Score at -3σ, and ensemble spread correlation with GEFS-Full, while columns are for fields (a) mean sea level pressure, (b) temperature at 2 meters, and (c) eastward wind speed at 850hPa.

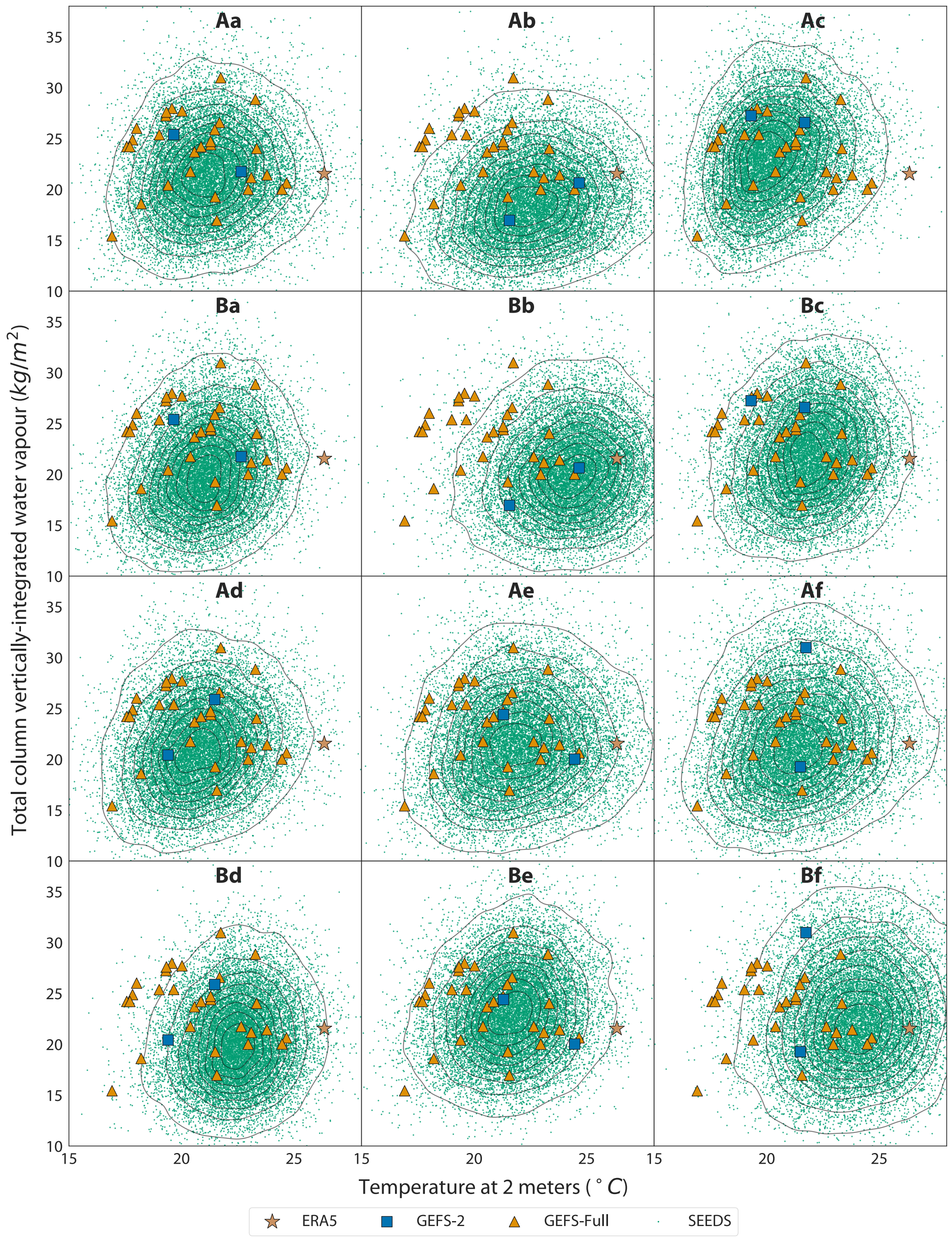

Fig. S13. Comparison between SEEDS-GEE and SEEDS-GPP from the same seeds. Each panel shows the temperature at 2 meters and total column water vapour of the grid point near Lisbon on 2022/07/14 from 16,384 SEEDS generated samples (shown as dots with kernel density level curves) conditioned on 2 random seeds GEFS-2 from 7-day lead time GEFS-Full. Panel rows (A) are from SEEDS-GEE while (B) SEEDS-GPP. Panels (a–f) are for 6 independent random 2-member seeds. The plots are paired vertically, for example, (Aa) and (Ba) have the same seeds for comparison.

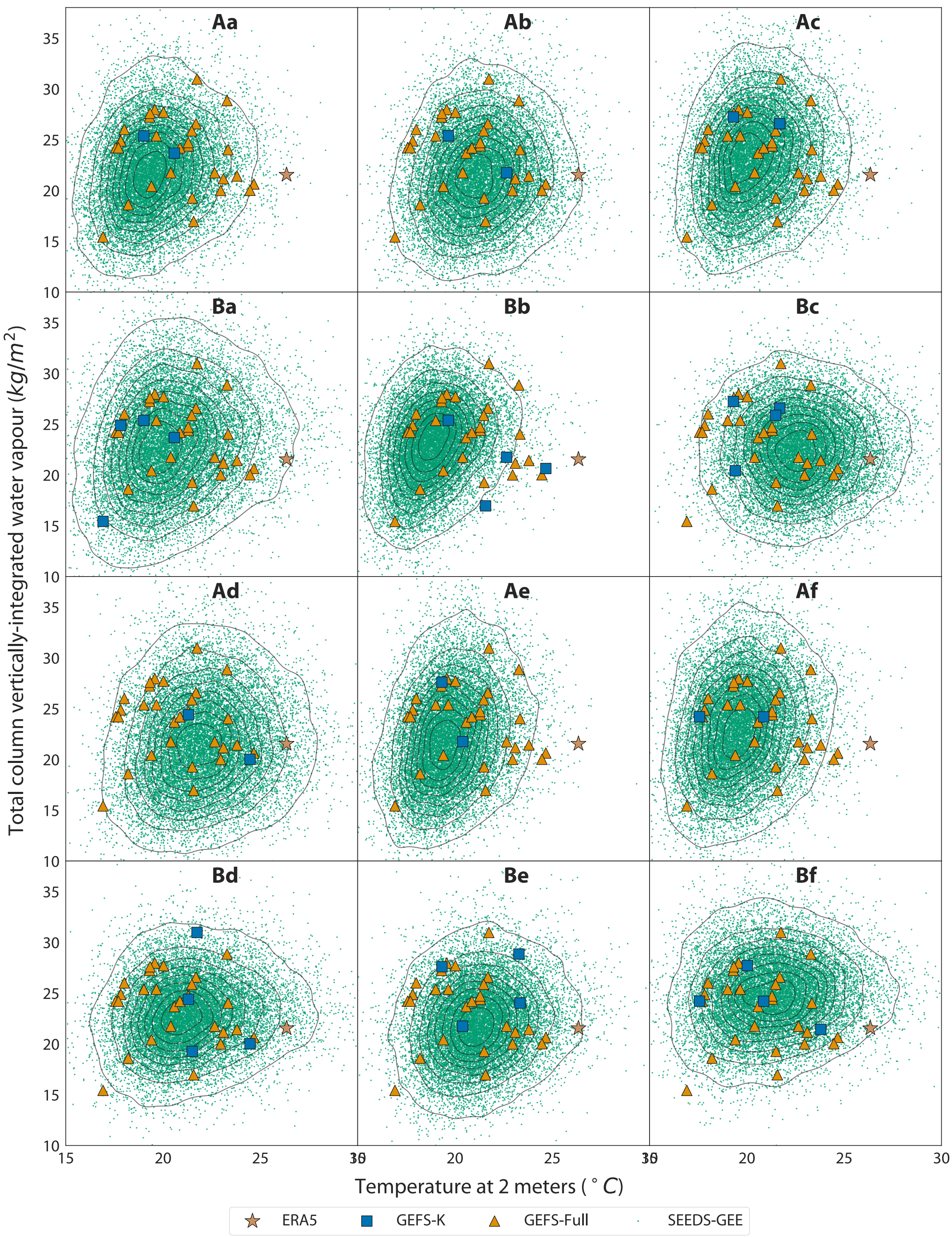

Fig. S14. Comparison between SEEDS-GEE with different number of seeds K. Each panel shows the temperature at 2 meters and total column water vapour of the grid point near Lisbon on 2022/07/14 from 16,384 SEEDS-GEE generated samples (shown as dots with kernel density level curves) conditioned on $K = 2, 4$ random seeds GEFS-K from 7-day lead time GEFS-Full. Panel rows (A) show samples with 2 seeds while (B) those with 4 seeds. Columns (a–f) are for 6 independent random draws of the seeds. The plots are paired vertically, for example, the 2 seeds in (Aa) are included in the 4 seeds in (Ba) for comparison.

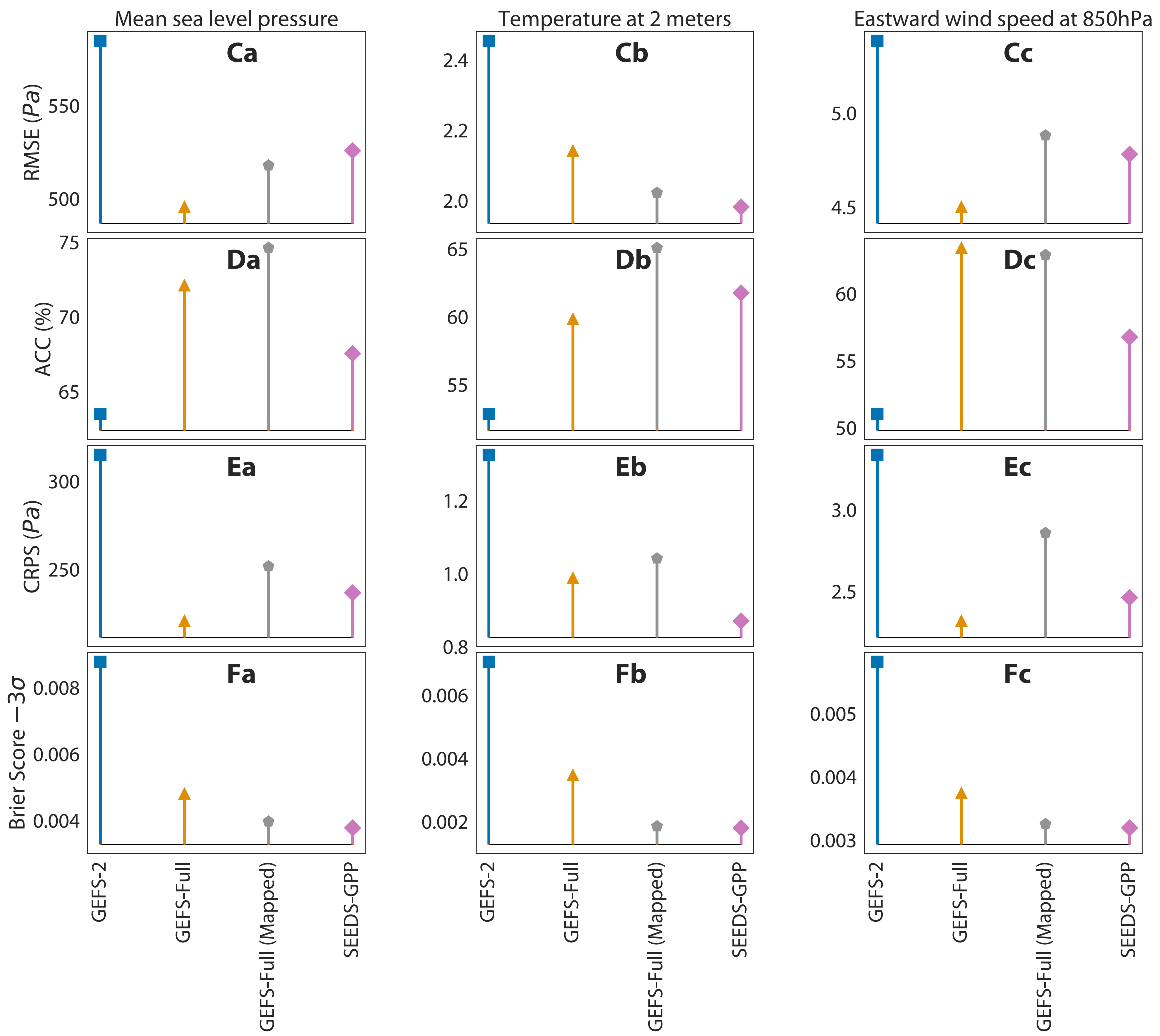

Fig. S15. Comparison of SEEDS-GPP with quantile-mapped GEFS-Full. Panel rows (C–F) show the RMSE, ACC, CRPS, and Brier Score at -3σ of different ensembles for year 2022. GEFS-Full (Mapped) is post-processed from GEFS-Full using pointwise isotonic regression based on 20 years of GEFS reforecasts (GEFS-RF5) and the ERA5 climatology. Columns are for (a) mean sea level pressure, (b) temperature at 2 meters, and (c) eastward wind speed at 850hPa. All metrics are computed for 7-day forecasts. SEEDS-GPP ensembles have 512 members generated from 2 random seeds (GEFS-2) of GEFS-Full. Metrics are computed using ERA5 as ground truth, and for issue times in 2022.

REFERENCES AND NOTES

1. P. Bauer, A. Thorpe, G. Brunet, The quiet revolution of numerical weather prediction. *Nature* **525**, 47–55 (2015).

2. Y. Zhu, Z. Toth, R. Wobus, D. Richardson, K. Mylne, The economic value of ensemble-based weather forecasts. *Bull. Am. Meteorol. Soc.* **83**, 73–83 (2002).

3. T. Palmer, The ECMWF ensemble prediction system: Looking back (more than) 25 years and projecting forward 25 years. *Q. J. R. Meteorol. Soc.* **145**, 12–24 (2019).

4. ECMWF, *IFS Documentation CY47R3 - Part V Ensemble Prediction System* (ECMWF, 2021); www.ecmwf.int/node/20199.

5. X. Zhou, Y. Zhu, D. Hou, B. Fu, W. Li, H. Guan, E. Sinsky, W. Kolczynski, X. Xue, Y. Luo, J. Peng, B. Yang, V. Tallapragada, P. Pegion, The development of the NCEP global ensemble forecast system version 12. *Weather Forecast.* **37**, 1069–1084 (2022).

6. M. Leutbecher, Ensemble size: How suboptimal is less than infinity? *Q. J. R. Meteorol. Soc.* **145**, 107–128 (2019).

7. T. N. Palmer, The economic value of ensemble forecasts as a tool for risk assessment: From days to decades. *Q. J. R. Meteorol. Soc.* **128**, 747–774 (2002).

8. J. Lin, K. Emanuel, J. L. Vigh, Forecasts of hurricanes using large-ensemble outputs. *Weather Forecast.* **35**, 1713–1731 (2020).

9. F. Vitart, C. Cunningham, M. DeFlorio, E. Dutra, L. Ferranti, B. Golding, D. Hudson, C. Jones, C. Lavaysse, J. Robbins, M. K. Tippett, Chapter 17 - Sub-seasonal to seasonal prediction of weather extremes, in *Sub-Seasonal to Seasonal Prediction* (Elsevier, 2019), pp. 365–386.

10. E. M. Fischer, U. Beyerle, L. Bloin-Wibe, C. Gessner, V. Humphrey, F. Lehner, A. G. Pendergrass, S. Sippel, J. Zeder, R. Knutti, Storylines for unprecedented heatwaves based on ensemble boosting. *Nat. Commun.* **14**, 4643 (2023).

11. E. Bevacqua, L. Suarez-Gutierrez, A. Jézéquel, F. Lehner, M. Vrac, P. Yiou, J. Zscheischler, Advancing research on compound weather and climate events via large ensemble model simulations. *Nat. Commun.* **14**, 2145 (2023).

12. R. Buizza, Introduction to the special issue on "25 years of ensemble forecasting". *Q. J. R. Meteorol. Soc.* **145**, 1–11 (2019).

13. C. Mass, The uncoordinated giant II: Why U.S. operational numerical weather prediction is still lagging and how to fix It. *Bull. Am. Meteorol. Soc.* **104**, E851–E871 (2023).

14. P. Dhariwal, A. Nichol, Diffusion models beat GANs on image synthesis. *Adv. Neural Inf. Process.* **34**, 8780–8794 (2021).

15. J. Ho, W. Chan, C. Saharia, J. Whang, R. Gao, A. Gritsenko, D. P. Kingma, B. Poole, M. Norouzi, D. J. Fleet, T. Salimans, Imagen video: High definition video generation with diffusion models. arXiv:2210.02303 [cs.CV] (5 October 2022).

16. R. Rombach, A. Blattmann, D. Lorenz, P. Esser, B. Ommer, High-resolution image synthesis with latent diffusion models, in *Proceedings of the IEEE Conference on Computer Vision and Pattern Recognition (CVPR)* (IEEE, 2022), pp. 10684–10695; https://github.com/CompVis/latent-diffusion.

17. J. Leinonen, U. Hamann, D. Nerini, U. Germann, G. Franch, Latent diffusion models for generative precipitation nowcasting with accurate uncertainty quantification. arXiv:2304.12891 [physics.ao-ph] (25 April 2023).

18. Z. Gao, X. Shi, B. Han, H. Wang, X. Jin, D. Maddix, Y. Zhu, M. Li, Y. Wang, PreDiff: Precipitation nowcasting with latent diffusion models. arXiv:2307.10422 [cs.LG] (28 December 2023).

19. H. Addison, E. Kendon, S. Ravuri, L. Aitchison, P. Watson, Machine learning emulation of a local-scale UK climate model, in *NeurIPS 2022 Workshop on Tackling Climate Change with Machine Learning* (Climate Change AI, 2022); www.climatechange.ai/papers/neurips2022/21/paper.pdf.

20. S. Bassetti, B. Hutchinson, C. Tebaldi, B. Kravitz, DiffESM: Conditional emulation of Earth system models with diffusion models. arXiv:2304.11699 [physics.ao-ph] (23 April 2023).

21. H. Hersbach, B. Bell, P. Berrisford, S. Hirahara, A. Horányi, J. Muñoz-Sabater, J. Nicolas, C. Peubey, R. Radu, D. Schepers, A. Simmons, C. Soci, S. Abdalla, X. Abellan, G. Balsamo, P. Bechtold, G. Biavati, J. Bidlot, M. Bonavita, G. De Chiara, P. Dahlgren, D. Dee, M. Diamantakis, R. Dragani, J. Flemming, R. Forbes, M. Fuentes, A. Geer, L. Haimberger, S. Healy, R. J. Hogan, E. Hólm, M. Janisková, S. Keeley, P. Laloyaux, P. Lopez, C. Lupu, G. Radnoti, P. de Rosnay, I. Rozum, F. Vamborg, S. Villaume, J.-N. Thépaut, The ERA5 global reanalysis. *Q. J. R. Meteorol. Soc.* **146**, 1999–2049 (2020).

22. S. Scher, G. Messori, Predicting weather forecast uncertainty with machine learning. *Q. J. R. Meteorol. Soc.* **144**, 2830–2841 (2018).

23. M. A. Sacco, J. J. Ruiz, M. Pulido, P. Tandeo, Evaluation of machine learning techniques for forecast uncertainty quantification. *Q. J. R. Meteorol. Soc.* **148**, 3470–3490 (2022).

24. R. Brecht, A. Bihlo, Computing the ensemble spread from deterministic weather predictions using conditional generative adversarial networks. *Geophys. Res. Lett.* **50**, e2022GL101452 (2023).

25. P. Isola, J. Zhu, T. Zhou, A. A. Efros, Image-to-image translation with conditional adversarial networks, in *2017 IEEE Conference on Computer Vision and Pattern Recognition (CVPR)* (IEEE, 2017), pp. 5967–5976; http://doi.ieeecomputersociety.org/10.1109/CVPR.2017.632.

26. T. S. Finn, Self-attentive ensemble transformer: Representing ensemble interactions in neural networks for earth system models. arXiv:2106.13924 [cs.LG] (10 July 2021).

27. P. Grönquist, C. Yao, T. Ben-Nun, N. Dryden, P. Dueben, S. Li, T. Hoefler, Deep learning for post-processing ensemble weather forecasts. *Philos. Trans. A Math. Phys. Eng. Sci.* **379**, 20200092 (2021).

28. L. Harris, A. T. T. McRae, M. Chantry, P. D. Dueben, T. N. Palmer, A generative deep learning approach to stochastic downscaling of precipitation forecasts. *J. Adv. Model. Earth Syst.* **14**, e2022MS003120 (2022).

29. S. Scher, S. Jewson, G. Messori, Robust worst-case scenarios from ensemble forecasts. *Weather Forecast.* **36**, 1357–1373 (2021).

30. H. Guan, Y. Zhu, E. Sinsky, B. Fu, W. Li, X. Zhou, X. Xue, D. Hou, J. Peng, M. M. Nageswararao, V. Tallapragada, T. M. Hamill, J. S. Whitaker, G. Bates, P. Pegion, S. Frederick, M. Rosencrans, A. Kumar, GEFSv12 reforecast dataset for supporting subseasonal and hydrometeorological applications. *Mon. Weather Rev.* **150**, 647–665 (2022).

31. E. N. Lorenz, Atmospheric predictability experiments with a large numerical model. *Tellus* **34**, 505–513 (1982).

32. R. P. Worsnop, M. Scheuerer, T. M. Hamill, J. K. Lundquist, Generating wind power scenarios for probabilistic ramp event prediction using multivariate statistical post-processing. *Wind Energy Sci.* **3**, 371–393 (2018).

33. A. Witze, Extreme heatwaves: Surprising lessons from the record warmth. *Nature* **608**, 464–465 (2022).

34. A. Sánchez-Benítez, R. García-Herrera, D. Barriopedro, P. M. Sousa, R. M. Trigo, June 2017: The earliest European summer mega-heatwave of reanalysis period. *Geophys. Res. Lett.* **45**, 1955–1962 (2018).

35. D. S. Wilks, *Statistical Methods in the Atmospheric Sciences* (Elsevier, ed. 4th, 2019).

36. J. L. Anderson, A method for producing and evaluating probabilistic forecasts from ensemble model integrations. *J. Clim.* **9**, 1518–1530 (1996).

37. O. Talagrand, R. Vautard, B. Strauss, Evaluation of probabilistic prediction systems, in *Workshop on Predictability, 20–22 October 1997* (ECMWF, Shinfield Park, Reading, 1997), pp. 1–26.

38. G. Candille, O. Talagrand, Evaluation of probabilistic prediction systems for a scalar variable. *Q. J. R. Meteorol. Soc.* **131**, 2131–2150 (2005).

39. M. Bonavita, On the limitations of data-driven weather forecasting models. arXiv:2309.08473 [stat.ML] (3 November 2023).

40. Z. Ben-Bouallegue, J. A. Weyn, M. C. A. Clare, J. Dramsch, P. Dueben, M. Chantry, Improving medium-range ensemble weather forecasts with hierarchical ensemble transformers. arXiv:2303.17195 [physics.ao-ph] (20 October 2023).

41. J. Ma, Y. Zhu, R. Wobus, P. Wang, An effective configuration of ensemble size and horizontal resolution for the NCEP GEFS. *Adv. Atmos. Sci.* **29**, 782–794 (2012).

42. F. Vitart, Y. Takaya, Lagged ensembles in sub-seasonal predictions. *Q. J. R. Meteorol. Soc.* **147**, 3227–3242 (2021).

43. R. Hagedorn, T. M. Hamill, J. S. Whitaker, Probabilistic forecast calibration using ECMWF and GFS ensemble reforecasts. Part I: Two-meter temperatures. *Mon. Weather Rev.*. **136**, 2608–2619 (2008).

44. L. S. R. Froude, L. Bengtsson, K. I. Hodges, The prediction of extratropical storm tracks by the ECMWF and NCEP ensemble prediction systems. *Mon. Weather Rev.* **135**, 2545–2567 (2007).

45. C. Deser, F. Lehner, K. B. Rodgers, T. Ault, T. L. Delworth, P. N. DiNezio, A. Fiore, C. Frankignoul, J. C. Fyfe, D. E. Horton, J. E. Kay, R. Knutti, N. S. Lovenduski, J. Marotzke, K. A. McKinnon, S. Minobe, J. Randerson, J. A. Screen, I. R. Simpson, M. Ting, Insights from Earth system model initial-condition large ensembles and future prospects. *Nat. Clim. Chang.* **10**, 277–286 (2020).

46. WMO, *Guidelines on Ensemble Prediction System Postprocessing* (WMO-No. 1254, World Meteorological Organization, 2021).

47. D. A. Lavers, A. Simmons, F. Vamborg, M. J. Rodwell, An evaluation of ERA5 precipitation for climate monitoring. *Q. J. R. Meteorol. Soc.* **148**, 3152–3165 (2022).

48. C. Ronchi, R. Iacono, P. S. Paolucci, The “Cubed Sphere”: A new method for the solution of partial differential equations in spherical geometry. *J. Comput. Phys.* **124**, 93–114 (1996).

49. M. Dabernig, G. J. Mayr, J. W. Messner, A. Zeileis, Spatial ensemble post-processing with standardized anomalies. *Q. J. R. Meteorol. Soc.* **143**, 909–916 (2017).

50. W. Qian, J. Du, Y. Ai, A review: Anomaly-based versus full-field-based weather analysis and forecasting. *Bull. Am. Meteorol. Soc.* **102**, E849–E870 (2021).

51. I. Lopez-Gomez, A. McGovern, S. Agrawal, J. Hickey, Global extreme heat forecasting using neural weather models. *Artif. Intell. Earth Syst.* **2**, e220035 (2023).

52. J. Ho, A. Jain, P. Abbeel, Denoising diffusion probabilistic models. *Adv. Neural Inf. Process.* **33**, 6840–6851 (2020).

53. Y. Song, J. Sohl-Dickstein, D. P. Kingma, A. Kumar, S. Ermon, B. Poole, Score-based generative modeling through stochastic differential equations, in *International Conference on Learning Representations* (2020).

54. A. Dosovitskiy, L. Beyer, A. Kolesnikov, D. Weissenborn, X. Zhai, T. Unterthiner, M. Dehghani, M. Minderer, G. Heigold, S. Gelly, J. Uszkoreit, N. Houlsby, An image is worth 16x16 words: Transformers for image recognition at scale, in *International Conference on Learning Representations* (2021).

55. J. Ho, N. Kalchbrenner, D. Weissenborn, T. Salimans, Axial attention in multidimensional transformers. arXiv:1912.12180 [cs.CV] (20 December 2019).

56. H. Hersbach, B. Bell, P. Berrisford, G. Biavati, A. Horányi, J. Muñoz Sabater, J. Nicolas, C. Peubey, R. Radu, I. Rozum, D. Schepers, A. Simmons, C. Soci, D. Dee, J-N. Thépaut, "ERA5 hourly data on single levels from 1940 to present," Copernicus Climate Change Service (C3S) Climate Data Store (CDS) (2023), https://doi.org/10.24381/cds.adbb2d47 [accessed 15 January 2023].

57. H. Hersbach, B. Bell, P. Berrisford, G. Biavati, A. Horányi, J. Muñoz Sabater, J. Nicolas, C. Peubey, R. Radu, I. Rozum, D. Schepers, A. Simmons, C. Soci, D. Dee, J-N. Thépaut, "ERA5 hourly data on pressure levels from 1940 to present," Copernicus Climate Change Service (C3S) Climate Data Store (CDS) (2023), https://doi.org/10.24381/cds.bd0915c6 [accessed 15 January 2023].

58. T. Karras, M. Aittala, T. Aila, S. Laine, Elucidating the design space of diffusion-based generative models. *Adv. Neural Inf. Process.* **35**, 26565–26577 (2022).

59. M. Tancik, P. Srinivasan, B. Mildenhall, S. Fridovich-Keil, N. Raghavan, U. Singhal, R. Ramamoorthi, J. Barron, R. Ng, Fourier features let networks learn high frequency functions in low dimensional domains. *Adv. Neural Inf. Process.* **33**, 7537–7547 (2020).

60. T. Gneiting, A. E. Raftery, Strictly proper scoring rules, prediction, and estimation. *J. Am. Stat. Assoc.* **102**, 359–378 (2007).

61. T. Dimitriadis, T. Gneiting, A. I. Jordan, Stable reliability diagrams for probabilistic classifiers. *Proc. Natl. Acad. Sci. U.S.A.* **118**, e2016191118 (2021).

62. G. W. Brier, Verification of forecasts expressed in terms of probability. *Mon. Weather Rev.* **78**, 1–3 (1950).

63. R. Benedetti, Scoring rules for forecast verification. *Mon. Weather Rev.* **138**, 203–211 (2010).

64. J. Slingo, T. Palmer, Uncertainty in weather and climate prediction. *Philos. Trans. A Math. Phys. Eng. Sci.* **369**, 4751–4767 (2011).

65. T. M. Hopson, P. J. Webster, A 1–10-day ensemble forecasting scheme for the major river basins of Bangladesh: Forecasting severe floods of 2003–07. *J. Hydrometeorol.* **11**, 618–641 (2010).
